# Associations of Radiomic Data Extracted from Static and Respiratory-Gated CT Scans with Disease Recurrence in Lung Cancer Patients Treated with SBRT

**DOI:** 10.1371/journal.pone.0169172

**Published:** 2017-01-03

**Authors:** Elizabeth Huynh, Thibaud P. Coroller, Vivek Narayan, Vishesh Agrawal, John Romano, Idalid Franco, Chintan Parmar, Ying Hou, Raymond H. Mak, Hugo J. W. L. Aerts

**Affiliations:** 1 Department of Radiation Oncology, Dana-Farber Cancer Institute, Brigham and Women’s Hospital, Harvard Medical School, Boston, United States of America; 2 Department of Radiology, Dana-Farber Cancer Institute, Brigham and Women’s Hospital, Harvard Medical School, Boston, United States of America; Gustave Roussy, FRANCE

## Abstract

Radiomics aims to quantitatively capture the complex tumor phenotype contained in medical images to associate them with clinical outcomes. This study investigates the impact of different types of computed tomography (CT) images on the prognostic performance of radiomic features for disease recurrence in early stage non-small cell lung cancer (NSCLC) patients treated with stereotactic body radiation therapy (SBRT). 112 early stage NSCLC patients treated with SBRT that had static free breathing (FB) and average intensity projection (AIP) images were analyzed. Nineteen radiomic features were selected from each image type (FB or AIP) for analysis based on stability and variance. The selected FB and AIP radiomic feature sets had 6 common radiomic features between both image types and 13 unique features. The prognostic performances of the features for distant metastasis (DM) and locoregional recurrence (LRR) were evaluated using the concordance index (CI) and compared with two conventional features (tumor volume and maximum diameter). P-values were corrected for multiple testing using the false discovery rate procedure. None of the FB radiomic features were associated with DM, however, seven AIP radiomic features, that described tumor shape and heterogeneity, were (CI range: 0.638–0.676). Conventional features from FB images were not associated with DM, however, AIP conventional features were (CI range: 0.643–0.658). Radiomic and conventional multivariate models were compared between FB and AIP images using cross validation. The differences between the models were assessed using a permutation test. AIP radiomic multivariate models (median CI = 0.667) outperformed all other models (median CI range: 0.601–0.630) in predicting DM. None of the imaging features were prognostic of LRR. Therefore, image type impacts the performance of radiomic models in their association with disease recurrence. AIP images contained more information than FB images that were associated with disease recurrence in early stage NSCLC patients treated with SBRT, which suggests that AIP images may potentially be more optimal for the development of an imaging biomarker.

## Introduction

Advances in science and technology have led to the understanding that each tumor, even within the same cancer type, has a myriad of distinct genotypic and phenotypic characteristics. This heterogeneity among tumors results in a spectrum of responses to treatments, and has led to the evolution of precision medicine [1]. In precision medicine, treatment plans are tailored towards the individual needs of each patient, largely based on their tumor characteristics and predicted therapeutic response, with the promise of improving overall survival and quality of life. The success of precision medicine relies on a means to capture the complexity and intrinsic properties of the tumor that is predictive of the most efficacious treatments. Radiomics is one method that aims to do this non-invasively by creating a quantitative portrayal of the tumor phenotype through the extraction of advanced imaging features from medical images [2–4]. These radiomic features describe the tumor phenotype through quantifying properties related to its shape, texture and image intensity, and have been predictive of clinical outcomes [5–15] and tumor characteristics, such as genotype and protein expression [16–18].

**Fig 1:**
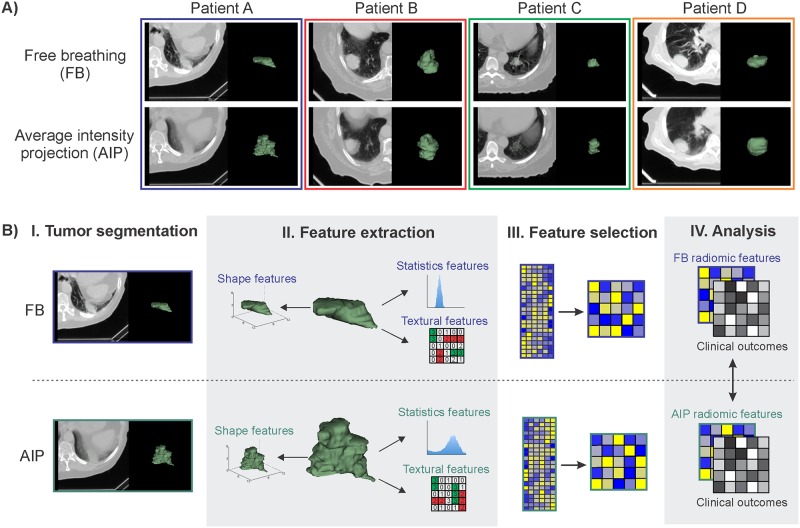
A) Examples of free breathing (FB) and average intensity projection (AIP) images, demonstrating the observable differences in tumor phenotype between each image type. AIP images were reconstructed from 4D computed tomography (CT) scans. B) Schematic representation of the radiomics workflow for FB and AIP images. I. CT images of the patient are acquired and the tumor is segmented. II. Imaging features (radiomic and conventional features) are extracted from the tumor volume. III. Radiomic features undergo a feature dimension reduction process to generate a low-dimensional feature set based on feature stability and variance. IV. Imaging features are then analyzed with clinical outcomes to evaluate their prognostic power. FB and AIP radiomics features are compared.

The majority of radiomics studies have focussed on investigating features extracted from a single image type. However, it is important to consider that the tumor phenotype and its behaviour may be uniquely captured in different types of images, even within the same imaging modality. For example, in radiation therapy treatment planning, computed tomography (CT) is the main imaging modality utilized, but different types of CT images are acquired to provide additional information for the treatment plan. Commonly, treatment plans are designed on static free breathing (FB) helical CT images, however, in cases where organ motion is a concern, such as with lung tumors, four-dimensional (4D) CT image datasets are also acquired. This is the case for early stage non-small cell lung cancer (NSCLC) patients that are treated with stereotactic body radiation therapy (SBRT) (Fig 1a). FB scans can provide additional information for contouring normal tissue structures and alignment of the patient with the radiation field. The treatment course is planned on 4DCT images. The utilization of both types of CT scans is one factor that has contributed to the excellent survival and local control of NSCLC patients treated with SBRT [19–25].

In the field of radiomics, the impact of different types of images on the prognostic performance of radiomic models has not yet been thoroughly investigated. While previous studies have reported the influence of different image types, scanning parameters or reconstruction algorithms on the variation in feature values [26–29], not many studies have investigated the differences in prognostic performance of these radiomic features for clinical outcomes [30]. Early stage NSCLC patients treated with SBRT are an ideal cohort to evaluate the prognostic impact of different image types, as both FB and 4D CT scans are routinely acquired in the clinic for each patient. Furthermore, despite the successes of SBRT, 13–23% of these patients still experience recurrent disease [19–25] and radiomics may have an important role in identifying which patients would be at highest risk of recurrence in order to adapt their course of treatment with the addition or intensification of therapy [31]. However, with the multiple types of CT images readily available for these patients, it is unknown which type of image would be optimal for radiomic analysis. The aim of this current study is to perform an initial exploratory evaluation of the prognostic performance of radiomic features extracted from FB and 4D CT scans to potentially identify which image type contains the most predictive radiomic information for disease recurrence in SBRT patients (Fig 1B). Investigating the impact of image type on the prognostic performance of radiomic features is imperative for identifying the most optimal imaging biomarkers for precision medicine.

## Materials and Methods

### Patient characteristics

One hundred and seventy patients with early stage NSCLC that were treated with SBRT at our institution from 2009–2014 were included in this study. This study was Institutional Review Board (IRB) approved by the Dana-Farber Cancer Institute IRB. Waiver of consent was approved for this retrospective study. From these 170 patients, patients were excluded if they fulfilled any of the following criteria: did not have a FB CT on file (n = 10), the duration between the FB scan and beginning of SBRT was greater than 1 week (n = 2), had multiple SBRT treatment courses and/or multiple primary tumors (n = 17), received induction chemotherapy (n = 2), had metastases to the lung from other primary sites (n = 30), locally recurrent disease (n = 5), small cell lung cancer (n = 1) or atypical carcinoid (n = 1) histology, or overall stage III or IV (n = 1). Patients that did not complete the full course of treatment were also excluded (n = 1). After applying these exclusion criteria, 112 patients were included in the radiomics analysis. The patient, treatment and tumor characteristics are reported in Table 1. Details regarding SBRT treatment protocol and assessment of clinical outcomes can be found in S1 File.

**Table 1:** Patient, tumor, and treatment characteristics and clinical outcomes.

		Total (n = 112 patients) median (range) or number (%)
**Patient characteristics**		
Age		74 (47–89)
Gender	Female/ Male	57/ 55 (50.9/ 49.1)
Ethnicity	African-American	8 (7.1)
	Asian	2 (1.8)
	Caucasian	102 (91.1)
Smoking	Never/ Current/ Former	3/ 27/ 82 (2.7/ 24.1/ 73.2)
Pack-years		50 (0.4–180.0)
Performance status	0/ 1/ 2/ 3	17/ 50/ 39/ 6 (15.2/ 44.6/ 34.8/5.4)
**Tumor characteristics**		
Overall stage	IA/ IB/ IIA	94/ 17/ 1 (83.9/ 15.2/ 0.9)
T stage	T1a/ T1b/ T2a/ T2b	66/ 27/ 18/1 (58.9/ 24.1/ 16.1/ 0.9)
Histology	Adenocarcinoma	48 (42.8)
	Adenosquamous carcinoma	1 (0.9)
	Squamous cell carcinoma	27 (24.1)
	Undifferentiated NSCLC	16 (14.3)
	No pathology	20 (17.9)
**Treatment characteristics**		
SBRT technique	3D Conformal / VMAT	84/ 28 (75/ 25)
Prescribed radiation dose (Gy)		54 (48–60)
Radiation dose per fraction (Gy)		18 (10–18)
Number of radiation fractions	3/ 4/ 5	67/ 2/ 43 (59.8/ 1.8/ 38.4)
Delivered biologically effective dose (Gy)		151.2 (100–151.2)
**Clinical outcomes**		
Follow-up time (months)		20.8 (0.3–47.8)
Distant metastasis (DM)	No/ Yes	89/ 23 (79.5/ 20.5)
	Time to event (months)	10.0 (2.0–37.7)
	Estimate of freedom from DM at 2 years	74.0%
Locoregional recurrence (LRR)	No/ Yes	88/ 24 (78.6/ 21.4)
	Time to event (months)	8.8 (2.0–26.4)
	Estimate of freedom from LRR at 2 years	70.9%
Survival	No/ Yes	53/ 59 (47.3/ 52.7)
	Time to event (months)	22.5 (1.3–47.8)
	Estimate of survival at 2 years	61.8%

### CT image acquisition and tumor segmentation

All patients had both FB and 4D CT scans acquired on a GE LightSpeed RT16 CT scanner (GE Medical Systems, Milwaukee, WI, USA) according to standard clinical scanning protocols. The most common imaging slice thickness and pixel spacing was 2.5 mm and 1.27 mm by 1.27 mm, respectively. All FB and AIP images were acquired with 120 kVp, and a standard reconstruction convolution kernel. AIP images were reconstructed from 4D CT image datasets that were acquired in axial cine mode, corresponding to one breathing cycle. The primary tumor site was manually contoured on FB and AIP images by E.H., V.A., and Y.H. on Eclipse software (Varian Medical Systems, Palo Alto, CA, USA), and then individually verified by an expert radiation oncologist (R.H.M.).

### Radiomic feature extraction

A set of 644 radiomic features was extracted from tumor volumes isolated from FB or AIP images (Fig 1B) using an in-house Matlab 2013 toolbox (The Mathworks Inc., Natick, MA, USA) and 3D Slicer 4.4.0 software [32]. The intensities in the raw image were discretized using a bin width of 25 Hounsfield units for the texture features in order to increase sensitivity relative to the raw image, reduce image noise and normalize the intensities across all the patients. All CT voxels were resampled to 1 x 1 x 1 mm^3^ using a bicubic interpolation function prior to feature extraction. Radiomic features were categorized as shape, statistics or texture features. Shape features describe the three dimensional physical appearances of the tumor, statistics features quantify properties of the voxel intensity histogram, and texture features quantify the spatial relationships between voxel intensities. A brief description of the selected radiomic features can be found in S1 Table in the Supplementary Information and a full description of all the radiomic features can be found in the supplementary material from a previous study [5].

Radiomic features were compared against commonly used clinical CT metrics (referred to as “conventional features”), which included tumor volume and maximum diameter. The tumor diameter was calculated as the maximum diameter measured in a single axial imaging slice.

### Radiomic feature dimension reduction

All feature selection and statistical analyses was conducted using R software version 3.3.0 [33]. A two-step feature dimension reduction method was used to reduce the high-dimensional radiomic feature set to a low-dimensional radiomic feature set for analysis. First, stable features were selected using the test-retest Reference Image Database to Evaluate Therapy Response (RIDER) dataset [34]. The RIDER dataset consists of a series of CT images from 31 NSCLC patients obtained approximately 15 minutes apart in a similar position. 644 radiomic features were extracted from these images and were assessed for how well they correlated across the two sets of images by calculating the intraclass correlation coefficient (ICC) (using the “irr” package [35]). Features with an ICC greater than 0.8 were considered stable and selected for further analysis. This step reduced the number of features to 286 stable features.

Second, the set of stable features was further reduced to a set of features that would retain most of the variance within the data. Principal Component Analysis (PCA) and factor analysis was applied using the “FactoMineR” package [36]. Scores that retained 95% of the variability from the stable features and correlated by at least 99% to the PCA scores were selected. This resulted in 19 radiomic features for each image type (19 FB features, 19 AIP features). In total, 21 imaging features (19 radiomic and 2 conventional features) from each image type were investigated. The data for these features can be found in S2 File.

### Univariate data analysis

The correlations between the imaging features from the FB and AIP images were evaluated using the Spearman’s correlation coefficient (ρ). FB and AIP features that had a |ρ| > 0.8 were considered to have a strong correlation.

The feature values were normalized (centred and scaled) into z-scores. The association between imaging features and clinical outcomes (i.e. the difference in feature values between patients with and without an event) was analyzed at the median time of event (10 and 9 months for DM and LRR, respectively) using a two-sided Wilcoxon rank-sum test. The difference in feature values was calculated by determining the difference in the median feature values for patients with and without the event. Patients that were censored or did not have an event before the considered time point were excluded in the assessment.

The concordance index (CI) [37,38] was used as a measure of the prognostic power of the imaging features for the clinical outcomes and was calculated using the “survcomp” package version 1.16 from Bioconductor [39]. The CI is a generalization of the area under the receiver operating characteristic curve (AUC) that also incorporates time, and is a measure of the probability that between two randomly drawn samples, the sample with the higher value will have a higher probability of an event. A CI equal to 0.5 is equivalent to a random guess, greater than 0.5 indicates that the feature value is directly proportional to the probability of experiencing the outcome, and less than 0.5 indicates inverse proportionality (the lower the value, the higher the risk). P-values were computed using Noether’s test to determine the significance of the CI from random (CI = 0.5). Multiple testing correction was applied to all univariate results by the false discovery rate (FDR) procedure introduced by Benjamini and Hochberg [40], where p-values less than 0.05 were considered statistically significant.

### Multivariate data analysis

Five models for predicting DM were evaluated: FB conventional, FB radiomics, AIP conventional, AIP radiomics, and a combined FB and AIP radiomic model. A stratified cross validation approach was used, where the whole cohort was partitioned into training (80%) and validation (20%) datasets with matching event ratios. This stratified partitioning method was carried out 100 times, resulting in 100 different training (80%) and validation (20%) datasets. Multivariate models were trained on the training datasets and their performance was assessed in the corresponding validation dataset using concordance index (CI). In order to limit the number of features in each multivariate radiomics model, we used a lasso-based feature reduction method on the training dataset, which reduced the set of radiomic features to 5 features. The performance of each model was then assessed on the validation dataset. The performance between any two multivariate models was compared using a permutation test with 200 bootstrap iterations. P-values less than 0.05 were considered statistically significant. All the multivariate analysis was carried out using the R package “caret”.

## Results

The patient cohort included 112 early stage NSCLC patients that had been treated solely with SBRT. Patient and treatment characteristics are reported in Table 1. The patient cohort had a median age of 74 (range: 47–89), was approximately equally split between genders (50.9% female, 49.1% male), and predominantly Caucasian (91.1%) and former smokers (73.2%). All patients were overall stage I-II (N0, M0) and treated with SBRT with a median delivered biologically effective dose of 151.2 Gy (range: 100 Gy– 151.2 Gy). The median follow-up time was 20.8 months (range: 0.3–47.8 months). 20.5% (n = 23) of patients experienced DM with a median time to event of 10.0 months. 21.4% (n = 24) of patients developed LRR with a median time to event of 8.8 months. The 2-year estimates for DM and LRR were 74.0% and 70.9%, respectively. All patients had both FB and AIP images acquired. The mean number of slices per tumor in the AIP images was 10.8 slices (range: 1.1–26.6).

Radiomic features were extracted from both FB and AIP images from each patient. Each feature set was reduced to 19 radiomic features, which were selected based on stability and maintaining the variance in the feature datasets. Two sets (FB and AIP) of 21 imaging features (2 conventional, 19 radiomic) were included in our analysis (Table 2). The two feature sets shared six common radiomic features, 2 features describing tumor shape, 3 features describing the intensity histogram or statistics of the tumor, and 1 feature describing the homogeneity of the tumor texture. The remaining 13 radiomic features in both the FB and AIP feature sets were unique to each image type. The unique radiomic features to the FB images were statistics features (6 features) or texture features (7 features). The unique radiomic features to the AIP images belonged to all feature groups: shape (1 feature), statistics (4 features) and texture (8 features).

**Table 2:** Imaging features selected for analysis.

	*Feature Group*		*Feature*	
**Conventional features**	Conventional		Volume	
			Max diameter	
**Common features between FB and AIP images**	Shape		Sphericity	
			Sphere disproportionality	
	Statistics		Wv LLL max	
			Wv HHL range	
			LoG 3mm 2D skewness	
	Texture		LoG 3mm 3D GLCM homogeneity1	
**Unique features to FB and AIP images**	**FB**		**AIP**	
	*Feature Group*	*Feature*	*Feature Group*	*Feature*
	Statistics	Wv HLL max	Shape	Compactness2
		Wv LLH total energy	Statistics	LoG 3mm 3D skewness
		Wv LLH mean		Wv HHL kurtosis
		Wv LHL skewness		Wv LLH skewness
		Wv HLL var		Wv HLL skewness
		Wv HLH min	Texture	LoG 3mm 3D GLCM infoCorr2
	Texture	Wv LHL GLCM correl1		GLCM correl1
		Wv HLH GLCM correl1		LoG 3mm 3D GLCM correl1
		Wv LLL GLCM infoCorr2		Wv LLL GLCM clusShade
		LoG 3mm 3D GLCM clusProm		LoG 3mm 2D GLCM clus Prom
		Wv HLH GLSZM high intensity large area emphasis		Wv HLH RLGL low gray level run emphasis
		LoG 3mm 2D GLCM clusShade		Wv LHH GLSZM large area emphasis
		Wv LLL GLCM infoCorr1		Wv LHH GLCM correl1

Labels: Wv = wavelet; LoG = Laplacian of Gaussian; L = low; H = high; GLCM = Gray-Level Co-occurrence Matrix; GLSZM = Gray-Level Size Zone Matrix; RLGL = Run Low Gray Level;

The correlation between the FB and AIP imaging features was assessed using the Spearman’s correlation coefficient (ρ). The majority of the FB and AIP features were not strongly correlated (mean ρ = 0.0524) (S1 Fig), however, 12 pairs of FB and AIP features did have a very strong correlation (ρ > 0.8) (S2 Table). The FB and AIP features that were strongly correlated were shape (9 of the 12 pairs of correlated features) and texture features (3 of the 12 pairs).

**Fig 2:**
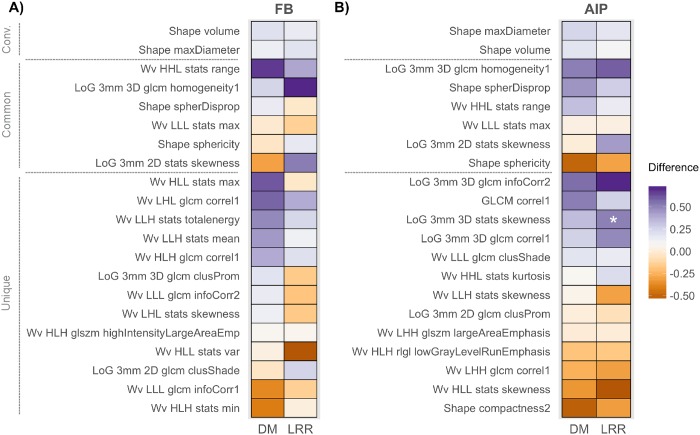
Heatmap of the association between imaging features and disease recurrence. Imaging features extracted from A) free breathing (FB) and B) average intensity projection (AIP) images were evaluated for their association with distant metastasis (DM) and locoregional recurrence (LRR). Features are grouped according to conventional (conv.) features, common features and unique features. “Common” features are radiomic features that had been selected from both FB and AIP images. “Unique” features are the radiomic features that were selected that are different between FB and AIP. The difference between the median values for each event status (event vs. no event) is plotted with the corresponding p-value indicated (Wilcoxon rank-sum test, FDR corrected p-values). The time point considered for DM and LRR was the median time of event (10 and 9 months for DM and LRR, respectively). *p-value < 0.05.

The association between the imaging feature values and clinical outcomes was investigated for LRR and DM (Fig 2, S2 and S3 Figs). None of the FB radiomic features were significantly associated with DM or LRR, however, one AIP radiomic feature describing the skewness of the intensity histogram (LoG 3mm 3D stats skewness), was significantly associated with LRR (p- value = 0.018). The difference between patients who had a LRR event versus those that did not was 0.51, indicating that higher values of skewness in the AIP images were associated with having a LRR event.

**Fig 3:**
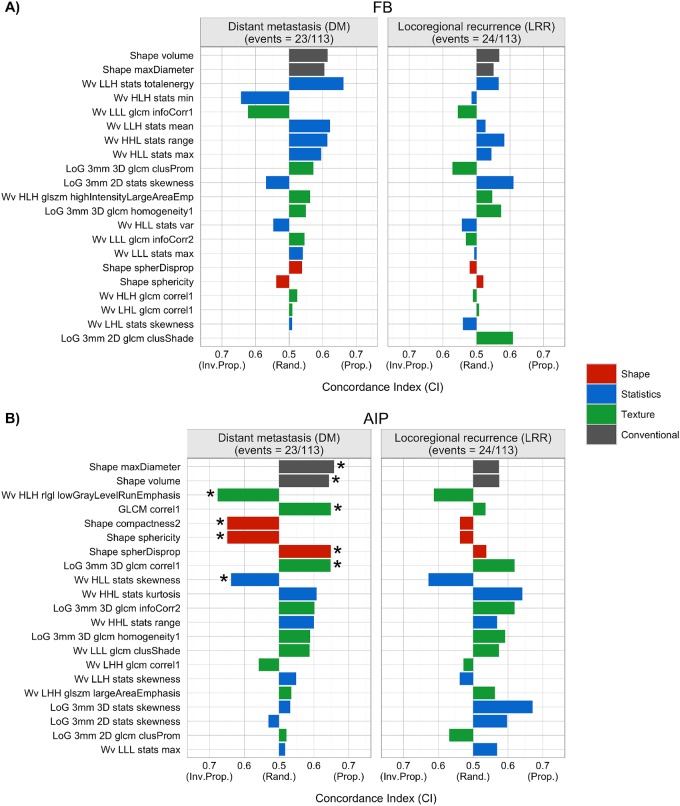
Prognostic performance of imaging features derived from A) FB or B) AIP images for disease recurrence in NSCLC patients treated with SBRT. Concordance indices (CI) are shown for each imaging feature and the clinical outcomes considered (distant metastasis (DM, left) and locoregional recurrence (LRR, right)). “Inv. Prop.”, “Rand.” and “Prop.” indicate inversely proportional, equivalent to a random guess, and directly proportional, respectively. Conventional features are shown in grey and radiomic features are shown in red (shape), blue (statistics), and green (texture). *p-value < 0.05 (Noether’s test, FDR corrected p-values).

The prognostic power of the FB and AIP imaging features was evaluated by calculating the concordance index (CI) for each feature (Fig 3, S3 Table). None of the FB conventional or radiomic features from this particular dataset were prognostic of DM or LRR. However, features extracted from these AIP images were prognostic of DM. Both conventional features, maximum tumor diameter and volume, were prognostic of DM with CIs of 0.658 and 0.643, respectively. Seven AIP radiomic features had CIs significantly greater than a random guess for DM that belonged to all feature groups: 3 texture features (Wavelet (Wv) High (H) Low (L)H Run Low Gray Level (RLGL), Low Gray Level Run emphasis (LGLRE), (Gray-Level Co-occurrence Matrix (GLCM) correl1 and Laplacian of Gaussian (LoG) 3mm 3D GLCM correl1, CI range: 0.648–0.676), 1 statistics feature (Wv HLL stats skewness, CI = 0.638), and 3 shape features (compactness, sphericity and sphere disproportionality had CIs of 0.648). The corresponding p-values for the significant features can be found in S3 Table. None of the AIP imaging features were prognostic of LRR.

**Fig 4:**
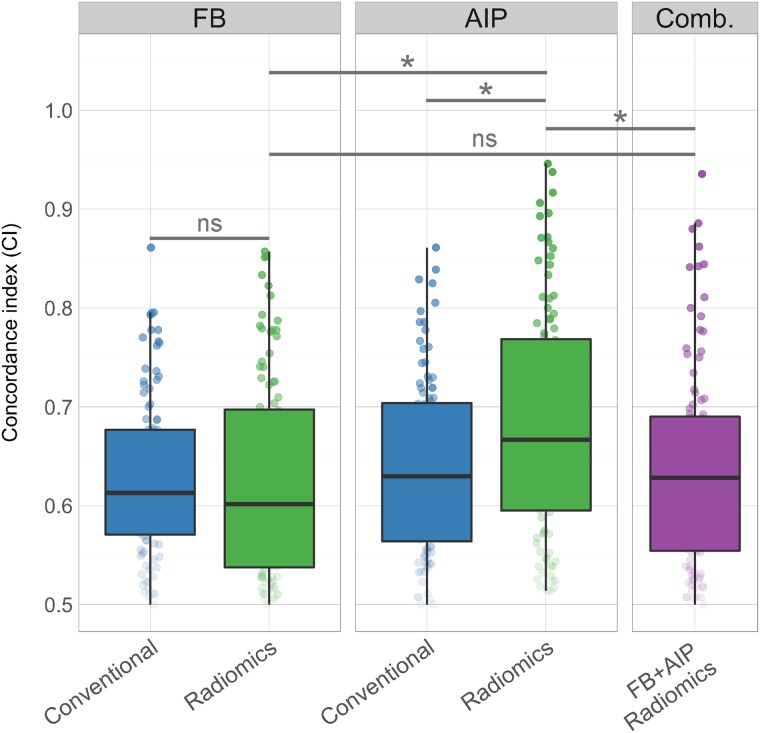
Performance of each multivariate model in predicting distant metastasis. Concordance indices are reported for the FB and AIP conventional and radiomic models, and a combined FB+AIP radiomics model, comparing the performance of each of model and image type. Cross validation was performed (80% training, 20% validation) to generate 100 models for each model type. Comb. Indicates the combined FB and AIP radiomics model. *p-value < 0.05; “ns” indicates not significant (p-value > 0.05).

Multivariate models were generated for DM based on the imaging features from FB and AIP images using cross validation. Two imaging models were generated (conventional and radiomics) for each image type (FB or AIP) and a combined radiomics model (Fig 4). The AIP radiomic model outperformed the FB radiomic model (CI = 0.667 for AIP, CI = 0.601 for FB, p-value = 0.025) and the AIP conventional model (CI = 0.630, p-value = 0.045). However, the FB radiomics model performed similarly to the FB conventional model (CI = 0.613, p-value = 0.575). Combining the FB and AIP radiomic models did not increase the prognostic performance greater than the AIP radiomic model alone (CI of combined model = 0.628; p-value = 0.01). The selected features for the FB and AIP radiomic multivariate models were relatively stable across each repetition (S4 and S5 Figs). The most commonly selected FB radiomic feature was Wv HLH stats min, which was selected for 84 out of the 100 models. Wv HLH GLSZM High Intensity Large Area Emphasis was chosen in 79 of the models and LoG 3mm 2D GLCM Clus Shade was chosen for 70 of the models. The most commonly selected AIP radiomic feature selected for multivariate AIP radiomic models was Wv LLL GLCM ClusShade, which was selected in 99 of the models. Wv HLH RLGL Low Gray Level Run Emphasis (LGLRE) was also chosen in 79 of the models and Wv HHL stats kurtosis was chosen in 75 of the models.

## Discussion

Radiomics may have a critical role in precision medicine as it quantitatively describes, with great detail, the tumor phenotype captured in medical images by applying advanced mathematical algorithms to generate a high-dimensional atlas of imaging features. The type of image used for radiomic feature extraction impacts the feature values [26], and therefore, it is important to evaluate the impact of these variations in the features on their association with and potential ability to predict clinical outcomes. Thus far, studies investigating the effect of image type on the prognostic performance of radiomic features may have been limited due to a lack of comparable cohorts with the same patient and treatment characteristics, and clinical outcomes. Early stage NSCLC patients treated with SBRT have both FB helical and 4D CT scans acquired as the standard of care. Thus, this cohort provides a direct comparison of the impact of image type on the prognostic performance of radiomic features, where the clinical data is identical for both image types. Many different reconstructions of 4D CT scans can be investigated, however, we chose to use the AIP over other reconstructions such as the maximum intensity projection (MIP). Clinically, AIP images are used for radiation therapy treatment planning, while MIP images are used for contouring the internal target volume. The MIP images may have more artifacts because they capture the extremes of tumor motion, which may impact radiomic features to a greater extent than averaging the intensity, as in AIP images. For this reason, we chose to use AIP images over MIP images for this analysis.

Nineteen radiomic features were selected for analysis from FB and AIP images. Notably, 13 of the 19 features were different between FB and AIP images. These features were selected based on maintaining the variance in the dataset, and therefore, the difference in feature sets indicates that the images contain different radiomics information and the image type impacts the feature values. Only one AIP radiomics feature was associated with LRR, however, none of the conventional or radiomics features from FB or AIP images were prognostic for LRR. Furthermore, none of the imaging features were associated with DM, although several features were prognostic for DM in our dataset. This highlights the important notion that although a feature may be associated with a clinical outcome, it may not necessarily be prognostic since the properties of the feature distribution that qualify a feature as associative are not the same as the properties that qualify a feature as prognostic [41].

The significant AIP radiomic features describe different aspects of the tumor phenotype captured in the CT image. The AIP texture feature, LGLRE, describes the distribution of low gray level intensity values, where a lower feature value indicates fewer regions of low gray level intensities. In our dataset, the CI was inversely proportional to the probability of an event, indicating that fewer regions of low gray level intensities were associated with a higher probability of developing DM. The AIP statistics feature, skewness, describes the shape of the voxel intensity histogram, where a lower skewness value indicates that the left tail (lower intensity values) of the intensity histogram is longer than the right tail (higher intensity values). Therefore, low skewness values indicate that there is a higher proportion of high intensity values, and in this dataset, is associated with a higher probability of DM. This directionality of the CI for skewness provides a complementary interpretation of LGLRE, which also found that fewer regions of low intensity values were associated with a higher probability of DM. The texture features GLCM correl1 and LoG 3mm GLCM correl1 describe the correlation of the gray level co-occurrence matrix, where the latter feature has a LoG filter applied, which emphasizes the areas in the image with a rapid intensity change. Lastly, the three shape features describe how similar and dissimilar the tumor shape is to a sphere (sphericity and sphere disproportionality, respectively) and how closely packed together the tumor shape is (compactness). Tumors that were less spherical and less compact were associated with a higher probability of developing DM.

Importantly, in our particular dataset, radiomic features extracted from AIP images were prognostic of DM (had CI significantly greater than 0.5), while radiomic features from FB images were not. This suggests that the tumor phenotype captured in FB and AIP images contain different prognostic information and the variation in feature values impacts the prognostic performance of the features. Importantly, despite having a strong correlation in FB and AIP images, the features describing the shape and dimensions of the tumor (shape radiomic features and conventional features) were prognostic in AIP images but not in FB. Furthermore, the three texture features (LGLRE and correl1) and one statistics feature (skewness) that were prognostic of DM in AIP images were not strongly correlated with any FB features. The difference in prognostic performance of the features between FB and AIP images highlights the impact of acquisition modes and reconstruction on the prognostic ability of the features since AIP images from 4D CT take into account organ motion, while FB images do not. Static FB CT scans represent a single snapshot of a dynamic lung tumor, and additionally, motion artifacts in FB scans can result in distortions of the tumor shape and compression of the tumor appearance in the image [42]. Furthermore, these artifacts impact the prognostic ability of the shape features in FB images since they may not be true depictions of the tumors. Motion artifacts are reduced in AIP images, which are reconstructions of multiple images across the breathing cycle, and thus, the tumors in these images may be a more accurate representation of the physical dimensions of the tumor, which may have a role in the prognostic ability of AIP shape features for DM.

Overall, we found that in our dataset, AIP images contained more prognostic radiomic features than FB images. This was also reflected in the multivariate analysis where radiomics models built from AIP radiomic features outperformed all other models (FB radiomic, and FB and AIP conventional). However, combining both FB and AIP radiomic models did not increase further increase their prognostic performance for DM, suggesting their radiomic information is not additive. Therefore, these results suggest that AIP images may be favoured over FB images for the development of imaging biomarkers for DM in NSCLC patients treated with SBRT, however, these particular conclusions pertain to our single dataset and requires further exploration and validation.

Previously, our group investigated the potential application of radiomics for lung cancer patients treated with SBRT using pre-treatment FB images and found that FB images did contain some prognostic information for predicting DM[9]. Comparatively, our current study uses a similar cohort of patients (112 patients in our current study vs. 113 patients previously), however the radiomics feature extraction and analysis is different. Our current study extracted a reduced set of radiomic features (644 features in the current study vs. 1605 features previously), which excluded many of the LoG features that were previously analyzed. Furthermore, we applied more stringent criteria for significance in our current study, where false discovery rate corrected p-values had a threshold of 0.05, whereas the previous studied applied a significance threshold of 0.1. Our previous study concluded that pre-treatment CT images may contain prognostic information for overall survival and DM. The current study elaborates on these findings and identifies that while FB images may contain prognostic information, the radiomics information found in AIP images may have stronger prognostic power than FB images and may potentially be a better option for developing an imaging biomarker for lung cancer patients treated with SBRT.

There are several limitations to our study that warrant discussion. SBRT is a fairly recently developed radiation therapy technique that is increasingly being adopted by more clinics and for more indications [43]. However, the recent implementation of SBRT limits the patient cohort size of our current study to only 113 patients that were treated between 2009 and 2014. As a result, our analytical methods were limited to an unsupervised feature selection method (not based on clinical outcomes) for radiomic feature dimension reduction and cross validation for the development and evaluation of multivariate models. However, we anticipate that over time, as clinical adoption of SBRT grows, a larger cohort of patients will be available for a validation study. Currently, SBRT is the standard of care for early stage medically inoperable NSCLC patients [44] and is being investigated as a non-invasive treatment option for operable early stage NSCLC patients [45]. Furthermore, the number of patients with early stage NSCLC is likely to increase due to lung screening efforts [46,47], and thus, radiomics applied for SBRT patients has great potential to impact a rapidly growing patient population of early stage NSCLC patients. Thus, with the availability of a larger patient cohort, future studies will validate these findings in larger cohorts and a single radiomics signature may be developed as an imaging biomarker for early stage NSCLC patients treated with SBRT to predict the risk of DM from AIP images.

Another limitation of our study was that it was restricted to a single institution, and thus, a single image acquisition protocol for both FB and 4D CT scans. Future investigations will be required to confirm our findings across multiple institutions and with external training and validation datasets to evaluate the generalizability of our findings to all early stage NSCLC SBRT patients. Despite these limitations, our current study demonstrates that different image types contain varying degrees of prognostic radiomics information and it is important to consider the type of image used for radiomics analysis and development of an imaging biomarker.

This study investigated the performance of radiomic features extracted from FB and AIP CT images in evaluating their associations with disease recurrence that may be predictive of outcome in early stage NSCLC patients who had been treated with SBRT. In our particular dataset, AIP images contained more prognostic radiomic features than FB images, and multivariate models built from AIP radiomic features had the highest performance compared to FB radiomic and conventional models. This study emphasizes the importance of selecting the appropriate image type for radiomic analysis and identifies that even within the same imaging modality (e.g. CT), some types of images contain more prognostic information than others. As the field of radiomics continues to evolve in its applications in precision medicine, the selection of an optimal image type for analysis is highly important to develop the best performing imaging biomarkers for clinical outcomes.

## Supporting Information

S1 Fig Heatmap of the Spearman’s correlation coefficient between FB and AIP imaging features. The imaging features included 2 conventional features and 19 radiomic features from FB and AIP images. Blue indicates a negative correlation, green indicates a positive correlation.
(PDF)

S2 Fig Boxplots of the distribution of feature values for patients with and without a distant metastasis (DM) event, calculated at 10 months. (PDF)

S3 Fig Boxplots of the distribution of feature values for patients with and without a locoregional recurrence (LRR) event, calculated at 9 months. (PDF)

S4 Fig Histogram of FB radiomic features chosen for each FB radiomic multivariate model across 100 iterations. (PDF)

S5 Fig Histogram of AIP radiomic features chosen for each AIP radiomic multivariate model across 100 iterations. (PDF)

S1 File Supplementary methods. (PDF)

S2 File Data. (PDF)

S1 Table Description of selected radiomic features. (PDF)

S2 Table Spearman’s correlation coefficient of strongly correlated FB and AIP imaging features. (PDF)

S3 Table Concordance indices of significant AIP imaging features. (PDF)
