# Supplementary material for: Associations of Radiomic Data Extracted from Static and Respiratory-Gated CT Scans with Disease Recurrence in Lung Cancer Patients Treated with SBRT
Source: PLoS One. 2017 Jan 3;12(1):e0169172. doi: 10.1371/journal.pone.0169172 (PMC5207741; doi:10.1371/journal.pone.0169172)
Supplement: S1 Fig — The imaging features included 2 conventional features and 19 radiomic features from FB and AIP images. Blue indicates a negative correlation, green indicates a positive correlation. (PDF) [file pone.0169172.s001.pdf]

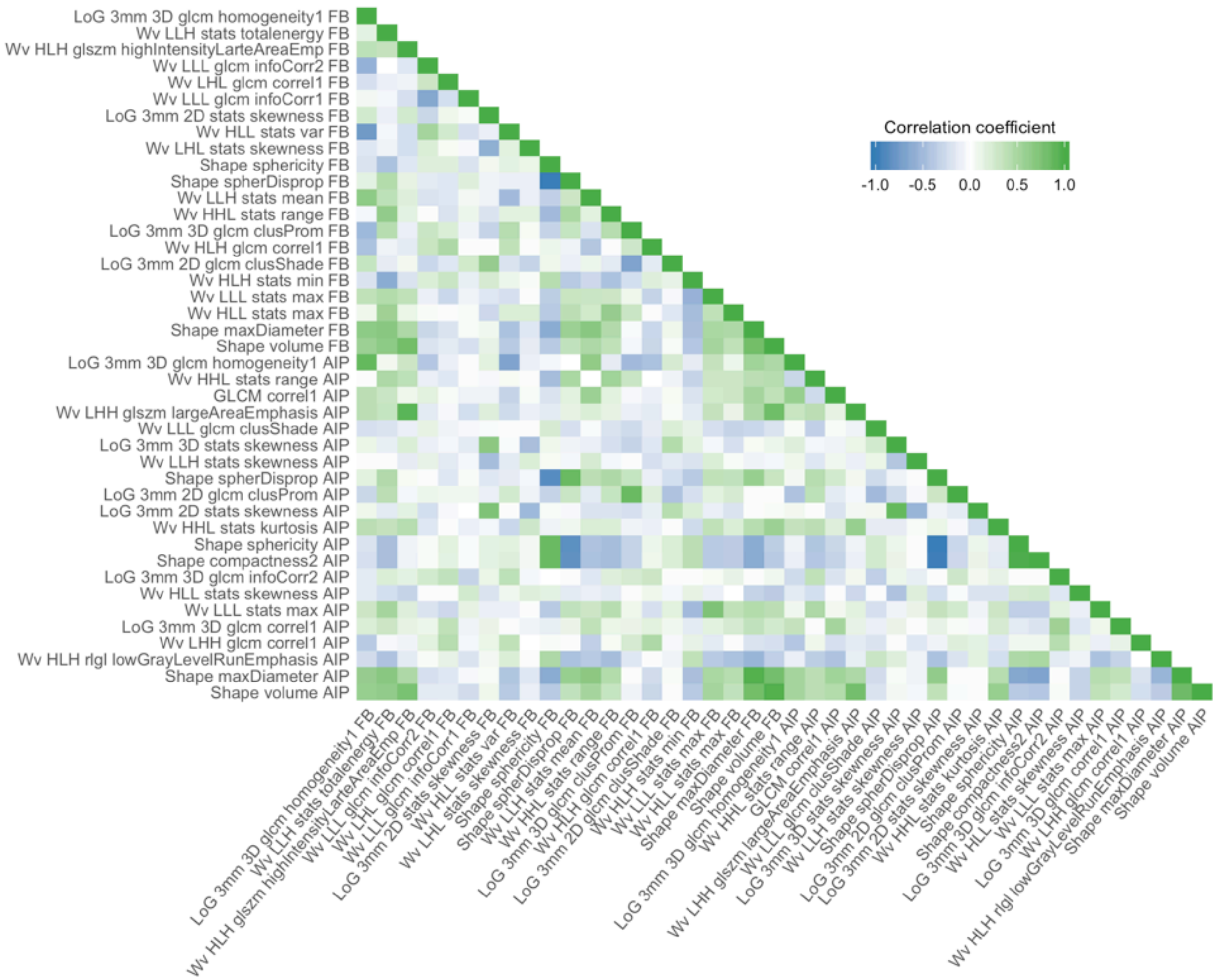


**S1 Fig.** Heatmap of the Spearman's correlation coefficient between FB and AIP imaging features. The imaging features included 2 conventional features and 19 radiomic features from FB and AIP images. Blue indicates a negative correlation, green indicates a positive correlation.
