# Supplementary material for: Associations of Radiomic Data Extracted from Static and Respiratory-Gated CT Scans with Disease Recurrence in Lung Cancer Patients Treated with SBRT
Source: PLoS One. 2017 Jan 3;12(1):e0169172. doi: 10.1371/journal.pone.0169172 (PMC5207741; doi:10.1371/journal.pone.0169172)
Supplement: S2 Fig — (PDF) [file pone.0169172.s002.pdf]

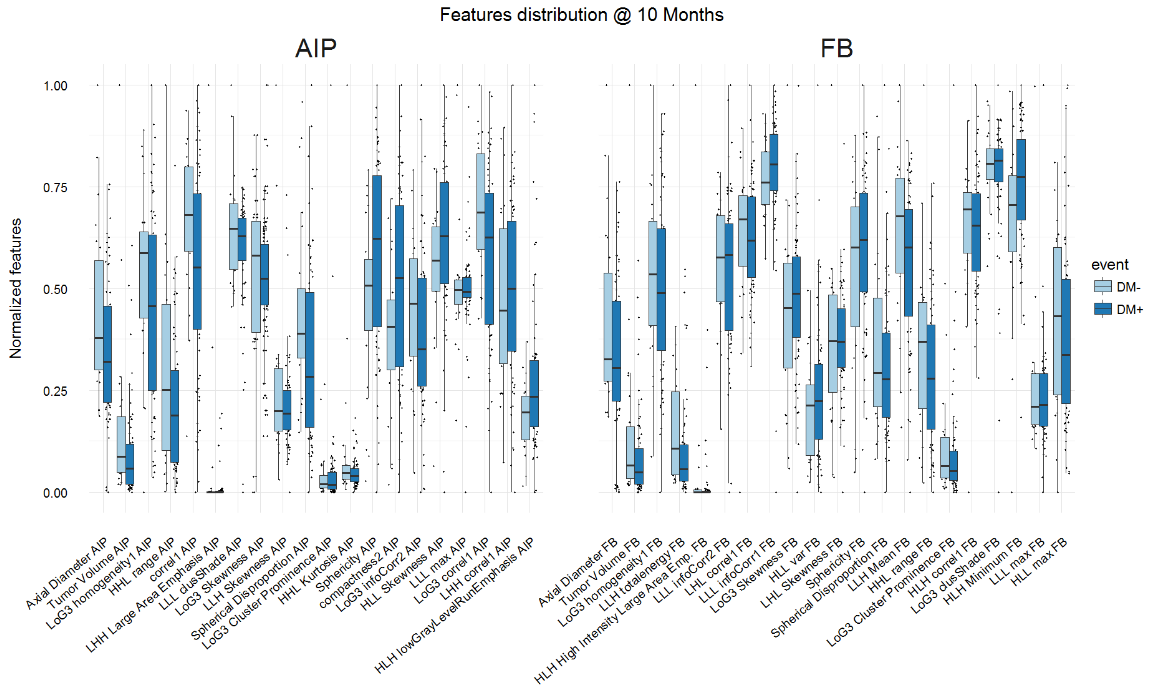

S2 Fig. Boxplots of the distribution of feature values for patients with and without a distant metastasis (DM) event, calculated at 10 months.
