# Supplementary material for: Associations of Radiomic Data Extracted from Static and Respiratory-Gated CT Scans with Disease Recurrence in Lung Cancer Patients Treated with SBRT
Source: PLoS One. 2017 Jan 3;12(1):e0169172. doi: 10.1371/journal.pone.0169172 (PMC5207741; doi:10.1371/journal.pone.0169172)
Supplement: S4 Fig — (PDF) [file pone.0169172.s004.pdf]

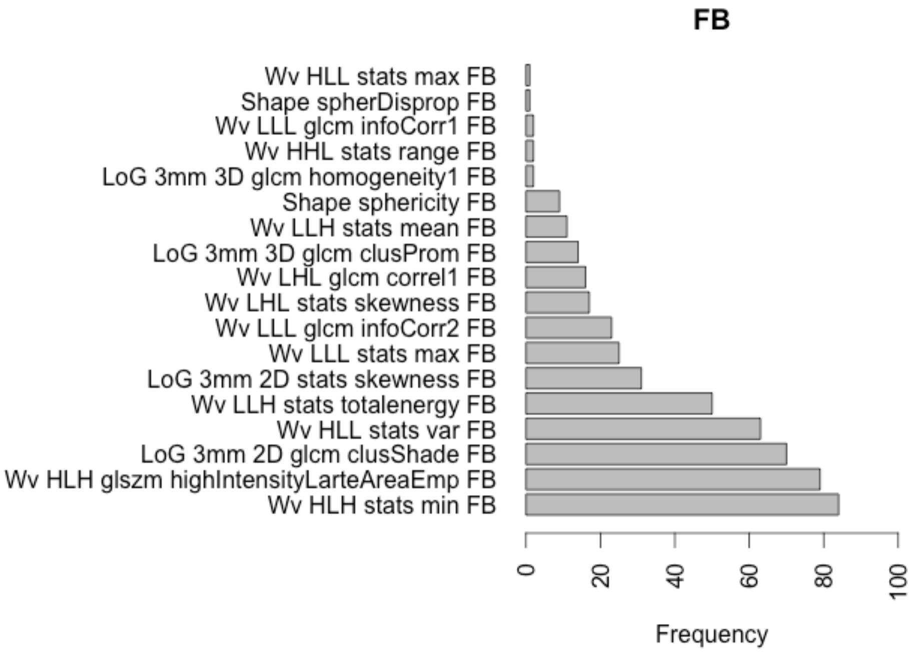

S4 Fig. Histogram of FB radiomic features chosen for each FB radiomic multivariate model across 100 iterations.
