# Supplementary material for: Associations of Radiomic Data Extracted from Static and Respiratory-Gated CT Scans with Disease Recurrence in Lung Cancer Patients Treated with SBRT
Source: PLoS One. 2017 Jan 3;12(1):e0169172. doi: 10.1371/journal.pone.0169172 (PMC5207741; doi:10.1371/journal.pone.0169172)
Supplement: S1 File — (PDF) [file pone.0169172.s006.pdf]

Supplementary Methods

SBRT treatment

All patients were treated with SBRT according to institutional standards and had peripheral tumors treated as defined in Radiation Therapy and Oncology Group (RTOG) 0236 (1). If the tumor motion was greater than 1 cm, abdominal compression was applied during treatment. Treatment planning was performed on 4D CT scans. The internal target volume (ITV) was defined on the maximum intensity projection (MIP) image. The planning target volume (PTV) was created on the MIP with a 5 mm margin from the ITV (no clinical target volume margin was applied). Patients received a dose of 10-12 Gy/fraction for 5 fractions for tumors in close proximity to the chest wall. All other tumors were treated with 12-14 Gy/fraction for 4 fractions or 18 Gy/fraction for 3 fractions. For daily set-up and image-guided treatment, Exac Trac, cone-beam CT and portal imaging using a linear accelerator were used.

Clinical outcomes

Clinical outcomes were assessed through follow-up contrast enhanced CT scans was performed every 3-6 months after SBRT to assess disease progression, based on the United States national guidelines (2). Details regarding the evaluation of clinical outcomes can be found in our previous study (3). Recurrence was also evaluated using positron emission tomography and/or biopsy. Contrast was not typically used in these patients with peripheral tumors. The clinical outcomes evaluated in our analysis were distant metastasis (DM) and locoregional recurrence (LRR). DM was considered as the spread of disease to sites outside of the lung, and LRR was considered as any recurrence that occurred locally, in the lobe of the lung and/or in the regional lymph nodes. 2-year estimates of survival and event-free probabilities were determined by the Kaplan-Meier method using the “survival” package in R (4).

References

1. Timmerman R, Paulus R, Galvin J, Michalski J, Straube W, Bradley J, et al. Stereotactic body radiation therapy for inoperable early stage lung cancer. Jama. 2010 Mar 17;303(11):1070-6. PubMed PMID: 20233825. Pubmed Central PMCID: 2907644.
2. Network NCC. NCCN guidelines : Non-Small Cell Lung Cancer (Version 7.2015)2015.
3. Mak RH, Hermann G, Lewis JH, Aerts HJ, Baldini EH, Chen AB, et al. Outcomes by tumor histology and KRAS mutation status after lung stereotactic body radiation therapy for early-stage non-small-cell lung cancer. Clinical lung cancer. 2015 Jan;16(1):24-32. PubMed PMID: 25450872. Pubmed Central PMCID: 4427190.
4. Therneau T. A Package for Survival Analysis in S. Version 2.38. 2015.
