# Supplementary material for: Associations of Radiomic Data Extracted from Static and Respiratory-Gated CT Scans with Disease Recurrence in Lung Cancer Patients Treated with SBRT
Source: PLoS One. 2017 Jan 3;12(1):e0169172. doi: 10.1371/journal.pone.0169172 (PMC5207741; doi:10.1371/journal.pone.0169172)
Supplement: S2 File — (PDF) [file pone.0169172.s007.pdf]

Data

## Outcome data

| Patient | DM | Time to DM (months) | LRR | Time to LRR (months) |
|---|---|---|---|---|
| 1 | 0 | | 0 | |
| 2 | 0 | | 0 | |
| 3 | 0 | | 0 | |
| 4 | 0 | | 0 | |
| 5 | 1 | 3.501388889 | 0 | |
| 6 | 0 | | 0 | |
| 7 | 0 | | 0 | |
| 8 | 0 | | 0 | |
| 9 | 1 | 4.031944444 | 1 | 4.031944444 |
| 10 | 1 | 7.498611111 | 1 | 7.498611111 |
| 11 | 1 | 3.665277778 | 1 | 3.665277778 |
| 12 | 0 | | 0 | |
| 13 | 0 | | 0 | |
| 14 | 0 | | 0 | |
| 15 | 0 | | 0 | |
| 16 | 1 | 8.934722222 | 1 | 8.934722222 |
| 17 | 0 | | 0 | |
| 18 | 0 | | 0 | |
| 19 | 0 | | 0 | |
| 20 | 0 | | 0 | |
| 21 | 0 | | 0 | |
| 22 | 0 | | 0 | |
| 23 | 0 | | 0 | |
| 24 | 0 | | 0 | |
| 25 | 0 | | 1 | 26.36805556 |
| 26 | 0 | | 0 | |
| 27 | 0 | | 0 | |
| 28 | 0 | | 0 | |
| 29 | 0 | | 0 | |
| 30 | 1 | 37.66666667 | 0 | |
| 31 | 1 | 10.89861111 | 1 | 7.398611111 |
| 32 | 1 | 8.998611111 | 1 | 8.998611111 |
| 33 | 0 | | 0 | |
| 34 | 1 | 20.86527778 | 1 | 20.86527778 |
| 35 | 0 | | 0 | |
| 36 | 0 | | 0 | |
| 37 | 1 | 5.633333333 | 1 | 3.566666667 |

| Patient | DM | Time to DM (months) | LRR | Time to LRR (months) |
|---|---|---|---|---|
| 38 | 0 | | 0 | |
| 39 | 1 | 2 | 1 | 2 |
| 40 | 1 | 11.53333333 | 0 | |
| 41 | 0 | | 0 | |
| 42 | 0 | | 0 | |
| 43 | 0 | | 0 | |
| 44 | 0 | | 0 | |
| 45 | 0 | | 0 | |
| 46 | 0 | | 0 | |
| 47 | 1 | 24.76666667 | 0 | |
| 48 | 0 | | 0 | |
| 49 | 1 | 8.366666667 | 1 | 8.366666667 |
| 50 | 0 | | 0 | |
| 51 | 0 | | 0 | |
| 52 | 0 | | 0 | |
| 53 | 0 | | 0 | |
| 54 | 0 | | 1 | 12.9 |
| 55 | 1 | 9.998611111 | 1 | 9.998611111 |
| 56 | 0 | | 0 | |
| 57 | 0 | | 0 | |
| 58 | 1 | 10.33333333 | 0 | |
| 59 | 1 | 13.93333333 | 1 | 6.8 |
| 60 | 0 | | 0 | |
| 61 | 0 | | 0 | |
| 62 | 0 | | 0 | |
| 63 | 0 | | 0 | |
| 64 | 0 | | 0 | |
| Patient | DM | Time to DM (months) | LRR | Time to LRR (months) |
| 65 | 1 | 13.7 | 1 | 13.7 |
| 66 | 0 | | 1 | 13.86666667 |
| 67 | 0 | | 0 | |
| 68 | 1 | 12.16666667 | 0 | |
| 69 | 0 | | 0 | |
| 70 | 0 | | 0 | |
| 71 | 0 | | 0 | |
| 72 | 0 | | 0 | |
| 73 | 0 | | 0 | |

| | | | | |
|---|---|---|---|---|
| 74 | 1 | 2.4 | 0 | |
| 75 | 1 | 5.631944444 | 1 | 5.631944444 |
| 76 | 0 | | 0 | |
| 77 | 0 | | 0 | |
| 78 | 0 | | 0 | |
| 79 | 0 | | 0 | |
| 80 | 0 | | 1 | 5.334722222 |
| 81 | 0 | | 1 | 15.73472222 |
| 82 | 0 | | 1 | 5.301388889 |
| 83 | 0 | | 0 | |
| 84 | 0 | | 0 | |
| 85 | 1 | 21.66666667 | 1 | 21.66666667 |
| 86 | 0 | | 0 | |
| 87 | 0 | | 0 | |
| 88 | 0 | | 0 | |
| 89 | 0 | | 0 | |
| 90 | 0 | | 0 | |
| 91 | 0 | | 0 | |
| 92 | 0 | | 0 | |
| 93 | 0 | | 0 | |
| 94 | 0 | | 0 | |
| 95 | 0 | | 0 | |
| 96 | 0 | | 0 | |
| 97 | 0 | | 0 | |
| 98 | 0 | | 0 | |
| 99 | 0 | | 0 | |
| 100 | 0 | | 0 | |
| 101 | 0 | | 1 | 18.96805556 |
| 102 | 0 | | 0 | |
| 103 | 0 | | 0 | |
| 104 | 0 | | 1 | 8.7 |
| 105 | 0 | | 0 | |
| 106 | 1 | 15.19861111 | 0 | |
| 107 | 0 | | 0 | |
| 108 | 0 | | 0 | |
| 109 | 0 | | 1 | 9.466666667 |
| 110 | 0 | | 0 | |
| 111 | 0 | | 0 | |
| 112 | 0 | | 0 | |

## Selected radiomic features

| Patient | LoG_sigma_3_mm_3D_glcm_homogeneity1_FB | Wavelet_LLH_stats_totalenergy_FB | Wavelet_HLH_glszm_highIntensityLarteAreaEmp_FB | Wavelet_LLL_glcm_infoCorr2_FB | Wavelet_LHL_glcm_correl1_FB | Wavelet_LLL_glcm_infoCorr1_FB |
|---|---|---|---|---|---|---|
| 1 | 0.34124 | 2926769.638 | 1917.92 | 0.98221 | 0.58166 | -0.51775 |
| 2 | 0.52854 | 13893824.88 | 2803556.692 | 0.88721 | 0.49898 | -0.24364 |
| 3 | 0.49226 | 6436958.533 | 8844.2941 | 0.89333 | 0.51187 | -0.2504 |
| 4 | 0.67229 | 93293610.63 | 5222295540 | 0.80495 | 0.38093 | -0.2901 |
| 5 | 0.62115 | 57948826.33 | 1942.725 | 0.85574 | 0.43788 | -0.28652 |
| 6 | 0.61058 | 32360830.56 | 231196070.7 | 0.84222 | 0.52759 | -0.25072 |
| 7 | 0.56634 | 5522652.116 | 2495001.903 | 0.79996 | 0.47614 | -0.20381 |
| 8 | 0.48401 | 22364476.05 | 44241.3478 | 0.86334 | 0.50128 | -0.23058 |
| 9 | 0.73673 | 69171403.57 | 13107134631 | 0.79324 | 0.47985 | -0.32202 |
| 10 | 0.64118 | 50070953.23 | 797182047.3 | 0.83125 | 0.27829 | -0.26941 |
| 11 | 0.52598 | 22449552.5 | 8170157.861 | 0.87871 | 0.50658 | -0.24741 |
| 12 | 0.50667 | 8300960.242 | 282500.0286 | 0.89069 | 0.44777 | -0.24905 |
| 13 | 0.64284 | 18253198.89 | 59789407.81 | 0.82953 | 0.48658 | -0.25034 |
| 14 | 0.53332 | 39835678.14 | 68817698.48 | 0.90611 | 0.54284 | -0.26587 |
| 15 | 0.53585 | 16800941.29 | 12723699.41 | 0.82219 | 0.462 | -0.20954 |
| 16 | 0.48334 | 16043986.95 | 1645506 | 0.90613 | 0.52313 | -0.26292 |
| 17 | 0.49335 | 18244952.24 | 16440.1944 | 0.87628 | 0.45763 | -0.23189 |
| 18 | 0.43743 | 76758432.45 | 4565944.825 | 0.88862 | 0.4987 | -0.24216 |
| 19 | 0.66635 | 7131807.356 | 55413951.38 | 0.79502 | 0.44381 | -0.23015 |
| 20 | 0.55172 | 5172306.777 | 2430746.69 | 0.85269 | 0.50293 | -0.24107 |
| 21 | 0.62518 | 7736437.339 | 31741437.96 | 0.86951 | 0.42568 | -0.26694 |
| 22 | 0.54583 | 12592918.92 | 41081272.24 | 0.82388 | 0.49016 | -0.21171 |
| 23 | 0.64766 | 33009933.33 | 5119.0588 | 0.80741 | 0.46971 | -0.25286 |
| 24 | 0.53552 | 16226615.6 | 4443523.973 | 0.87803 | 0.51933 | -0.24529 |
| 25 | 0.58822 | 36432399.88 | 1434.0789 | 0.87402 | 0.47741 | -0.25713 |
| 26 | 0.5582 | 8895032.86 | 13314648.25 | 0.87144 | 0.4632 | -0.24426 |
| 27 | 0.46481 | 78113137.16 | 18385555.86 | 0.91032 | 0.42263 | -0.25567 |
| 28 | 0.54108 | 15460714.43 | 4118.3095 | 0.85833 | 0.53407 | -0.23848 |
| 29 | 0.69214 | 78355701.59 | 4013208563 | 0.87601 | 0.54321 | -0.32427 |
| 30 | 0.57514 | 44813374.2 | 65613333.73 | 0.85111 | 0.49105 | -0.23881 |
| 31 | 0.60669 | 7460642.207 | 5383042.214 | 0.82961 | 0.50185 | -0.25801 |
| 32 | 0.51632 | 5597436.733 | 433822.0303 | 0.91809 | 0.44563 | -0.28632 |
| 33 | 0.66212 | 27026879.55 | 314089400 | 0.79078 | 0.54865 | -0.25217 |
| 34 | 0.44397 | 9800832.655 | 10200.8857 | 0.8953 | 0.57511 | -0.25864 |
| 35 | 0.53159 | 11958429.15 | 7561574 | 0.85723 | 0.49808 | -0.23796 |
| 36 | 0.67572 | 7078126.861 | 24381173 | 0.80368 | 0.48012 | -0.24766 |
| 37 | 0.56866 | 30500452.67 | 145015.8857 | 0.80978 | 0.50907 | -0.23163 |

| | | | | | | |
|---|---|---|---|---|---|---|
| 38 | 0.62819 | 12116059.46 | 40693021.15 | 0.87852 | 0.48632 | -0.27439 |
| 39 | 0.45336 | 188692458.2 | 122726869.2 | 0.9211 | 0.48349 | -0.27348 |
| 40 | 0.55142 | 7042844.353 | 1881439.679 | 0.90158 | 0.51683 | -0.28491 |
| 41 | 0.54548 | 10328012.2 | 4629548.129 | 0.86544 | 0.53009 | -0.23498 |
| 42 | 0.72046 | 12282378.09 | 115801621.5 | 0.8826 | 0.4161 | -0.32904 |
| 43 | 0.46877 | 6594975.004 | 775702.8 | 0.87519 | 0.46938 | -0.23502 |
| 44 | 0.48241 | 19924009.06 | 1242525.371 | 0.83875 | 0.50683 | -0.21008 |
| 45 | 0.59889 | 31875847.77 | 112806876.1 | 0.87833 | 0.46817 | -0.296 |
| 46 | 0.58834 | 3846831.963 | 1413602.542 | 0.82331 | 0.47053 | -0.21745 |
| 47 | 0.60239 | 21394795.36 | 69840477.43 | 0.88434 | 0.55118 | -0.27631 |
| 48 | 0.52305 | 18447526.65 | 845.8611 | 0.88421 | 0.45753 | -0.24159 |
| 49 | 0.37599 | 29575334.52 | 3688.8286 | 0.89157 | 0.52448 | -0.23247 |
| 50 | 0.58446 | 50047322.16 | 118423731.8 | 0.86637 | 0.4178 | -0.2616 |
| 51 | 0.58932 | 35395259 | 75279396.73 | 0.87113 | 0.50637 | -0.27125 |
| 52 | 0.42806 | 11124290.34 | 54943.1071 | 0.87737 | 0.55454 | -0.23488 |
| 53 | 0.45089 | 6693926.091 | 93614.4242 | 0.93204 | 0.50792 | -0.30598 |
| 54 | 0.68859 | 28820538.91 | 841558322.6 | 0.86351 | 0.45076 | -0.29323 |
| 55 | 0.5527 | 9079895.127 | 1603.9697 | 0.88964 | 0.50374 | -0.25163 |
| 56 | 0.50287 | 44041945.54 | 58839374.94 | 0.83927 | 0.52951 | -0.20743 |
| 57 | 0.44866 | 3249857.229 | 38536.8276 | 0.90998 | 0.51062 | -0.27361 |
| 58 | 0.63961 | 16781531.17 | 75967242.71 | 0.86128 | 0.42095 | -0.27674 |
| 59 | 0.5947 | 17878121.52 | 41598399.93 | 0.80096 | 0.48955 | -0.22245 |
| 60 | 0.51341 | 6465705.496 | 1358244.143 | 0.88308 | 0.51367 | -0.24991 |
| 61 | 0.42374 | 5146933.139 | 7868.2727 | 0.94212 | 0.61012 | -0.33175 |
| 62 | 0.52885 | 38055942.6 | 70553636.31 | 0.86114 | 0.44734 | -0.24234 |
| 63 | 0.45183 | 14030635.05 | 1127970.222 | 0.85048 | 0.49031 | -0.22514 |
| 64 | 0.43617 | 4897908.169 | 17270 | 0.94707 | 0.56961 | -0.32389 |
| 65 | 0.48148 | 5486872.271 | 51684.7273 | 0.87992 | 0.52652 | -0.2326 |
| 66 | 0.70881 | 48430578.74 | 1663117242 | 0.69698 | 0.47907 | -0.22257 |
| 67 | 0.66202 | 20158941.45 | 128633462.2 | 0.82683 | 0.51372 | -0.27051 |
| 68 | 0.50433 | 64271442.88 | 107459958.6 | 0.82894 | 0.39106 | -0.20861 |
| 69 | 0.5437 | 11757432.98 | 3209324.667 | 0.79812 | 0.43683 | -0.19015 |
| 70 | 0.59797 | 20532544.28 | 79557200.37 | 0.90774 | 0.53143 | -0.28839 |
| 71 | 0.43215 | 6103949.245 | 9398.303 | 0.86697 | 0.44564 | -0.22049 |
| 72 | 0.51537 | 21809605.93 | 12470362.07 | 0.91048 | 0.47929 | -0.26742 |
| 73 | 0.5972 | 21211249.84 | 31308758.41 | 0.86262 | 0.5487 | -0.2595 |
| 74 | 0.64942 | 77390035.93 | 1501000285 | 0.86758 | 0.55541 | -0.29412 |
| 75 | 0.50201 | 21567965.81 | 32104154.55 | 0.8552 | 0.43949 | -0.21904 |
| 76 | 0.57047 | 22964873.54 | 36282847.83 | 0.78024 | 0.44124 | -0.20427 |
| 77 | 0.4599 | 9784281.267 | 402362.1111 | 0.87048 | 0.39626 | -0.24056 |
| 78 | 0.41728 | 6596006.258 | 20247.5294 | 0.913 | 0.47712 | -0.27641 |

| | | | | | | |
|---|---|---|---|---|---|---|
| 79 | 0.46472 | 7566898.494 | 685817.1765 | 0.8885 | 0.4928 | -0.24689 |
| 80 | 0.65061 | 7423068.106 | 1952939.9 | 0.84882 | 0.56385 | -0.26944 |
| 81 | 0.60067 | 3474891.346 | 2971886.174 | 0.77015 | 0.54017 | -0.19434 |
| 82 | 0.43197 | 6512627.476 | 6666.697 | 0.92167 | 0.37218 | -0.31212 |
| 83 | 0.45062 | 4093298.576 | 3319.5938 | 0.90808 | 0.52493 | -0.27429 |
| 84 | 0.64895 | 2934763.136 | 6684895.765 | 0.70314 | 0.46066 | -0.17624 |
| 85 | 0.59313 | 17973756.56 | 8528.8667 | 0.74119 | 0.50052 | -0.2004 |
| 86 | 0.52119 | 103967516.4 | 95189560.29 | 0.8329 | 0.54069 | -0.20803 |
| 87 | 0.54506 | 24344947.76 | 2466.5806 | 0.80613 | 0.44781 | -0.19891 |
| 88 | 0.42528 | 13215362.68 | 462690.3947 | 0.85811 | 0.53759 | -0.20863 |
| 89 | 0.39791 | 1321643.169 | 2369.1429 | 0.97168 | 0.5461 | -0.46624 |
| 90 | 0.49377 | 3771382.456 | 368938.8846 | 0.90836 | 0.49729 | -0.2973 |
| 91 | 0.66106 | 73619165.67 | 1812011416 | 0.87749 | 0.4282 | -0.31959 |
| 92 | 0.49294 | 23749763.76 | 30562.7143 | 0.84181 | 0.49416 | -0.21791 |
| 93 | 0.51943 | 8681874.018 | 735825.5 | 0.84442 | 0.42101 | -0.21815 |
| 94 | 0.4033 | 6550213.186 | 126090.3333 | 0.81122 | 0.45886 | -0.18541 |
| 95 | 0.55475 | 9372946.274 | 5262237.9 | 0.86485 | 0.45911 | -0.26409 |
| 96 | 0.70894 | 23177521.86 | 1230562932 | 0.7834 | 0.40804 | -0.2418 |
| 97 | 0.58678 | 6488418.713 | 6225378.913 | 0.77354 | 0.44055 | -0.18143 |
| 98 | 0.51738 | 13515229.34 | 792906.9333 | 0.83481 | 0.51178 | -0.21073 |
| 99 | 0.57198 | 12867666.03 | 3925539.172 | 0.81717 | 0.47398 | -0.22881 |
| 100 | 0.48301 | 3009742.748 | 968.2333 | 0.89429 | 0.51901 | -0.25743 |
| 101 | 0.638 | 9579931.816 | 49436878.21 | 0.80084 | 0.5255 | -0.23469 |
| 102 | 0.46783 | 10081886.56 | 232619.2667 | 0.87482 | 0.41674 | -0.23212 |
| 103 | 0.54758 | 14382513.28 | 241176.9444 | 0.87756 | 0.50368 | -0.24797 |
| 104 | 0.58364 | 9587801.515 | 28441332.03 | 0.80643 | 0.46087 | -0.21732 |
| 105 | 0.44 | 100829537.4 | 6908 | 0.87337 | 0.47551 | -0.21484 |
| 106 | 0.53549 | 6329710.358 | 2300158.581 | 0.83576 | 0.48164 | -0.21947 |
| 107 | 0.6156 | 11489974.64 | 21652132.06 | 0.86868 | 0.481 | -0.277 |
| 108 | 0.42836 | 6331204.249 | 431585.3939 | 0.88028 | 0.44216 | -0.25357 |
| 109 | 0.56305 | 3906087.08 | 1561115.773 | 0.76537 | 0.43099 | -0.19971 |
| 110 | 0.52744 | 4443374.794 | 556911.5789 | 0.82684 | 0.43309 | -0.21867 |
| 111 | 0.5292 | 12895294.37 | 6815953.36 | 0.8012 | 0.45436 | -0.19302 |
| 112 | 0.51656 | 6216887.14 | 34914.2963 | 0.80812 | 0.40419 | -0.21895 |

| Patient | LoG_sigma_3_mm_2D_stats_skewness_FB | Wavelet_HLL_stats_var_FB | Wavelet_LHL_stats_skewness_FB | Shape_sphericity_FB | Shape_spherDisprop_FB | Wavelet_LLH_stats_mean_FB |
|---|---|---|---|---|---|---|
| 1 | -0.35786 | 2389.7668 | 0.094947 | 0.82513 | 1.2119 | -42.2154 |
| 2 | -0.11825 | 1336.1498 | -0.086517 | 0.8082 | 1.2373 | -12.9757 |
| 3 | 0.18338 | 2021.9045 | 0.071507 | 0.78966 | 1.2664 | -14.1226 |
| 4 | -0.011798 | 957.9104 | -0.66949 | 0.74913 | 1.3349 | -8.1312 |
| 5 | -0.7393 | 1771.9968 | 0.35904 | 0.58699 | 1.7036 | -4.5643 |
| 6 | -0.51484 | 1489.2765 | 0.1635 | 0.76838 | 1.3014 | -7.9585 |
| 7 | 0.39152 | 2872.6201 | -0.11417 | 0.64415 | 1.5524 | -18.0085 |
| 8 | -0.05761 | 2394.1956 | -0.066652 | 0.62845 | 1.5912 | -17.891 |
| 9 | 1.2273 | 544.4698 | -0.88495 | 0.66248 | 1.5095 | -5.3533 |
| 10 | -0.94008 | 853.6161 | 0.45303 | 0.68104 | 1.4683 | -1.6558 |
| 11 | -0.14903 | 1822.4386 | 0.17753 | 0.77885 | 1.2839 | -14.0369 |
| 12 | -0.23474 | 1530.8363 | 0.12154 | 0.79547 | 1.2571 | -4.8119 |
| 13 | 0.14993 | 878.0841 | -0.38969 | 0.66098 | 1.5129 | -8.1393 |
| 14 | -0.4534 | 2438.6697 | 0.30285 | 0.72854 | 1.3726 | -6.3867 |
| 15 | 0.014584 | 1289.6419 | -0.13935 | 0.79455 | 1.2586 | -11.6303 |
| 16 | 0.017463 | 2794.3268 | 0.42211 | 0.78982 | 1.2661 | -17.5273 |
| 17 | -0.053256 | 2353.9354 | 0.078027 | 0.7239 | 1.3814 | -16.6638 |
| 18 | -0.31956 | 3300.5424 | -0.2644 | 0.63414 | 1.5769 | -25.2399 |
| 19 | 0.20259 | 783.7509 | -0.13596 | 0.76485 | 1.3075 | -4.6474 |
| 20 | -0.094809 | 1840.7637 | -0.02608 | 0.85793 | 1.1656 | -19.458 |
| 21 | -0.6746 | 799.1042 | 0.4201 | 0.75285 | 1.3283 | -5.026 |
| 22 | -0.23098 | 1605.9113 | 0.27137 | 0.71603 | 1.3966 | -11.0879 |
| 23 | 0.48659 | 741.8864 | -0.34117 | 0.72447 | 1.3803 | -13.6236 |
| 24 | -1.14 | 2164.9433 | 0.56716 | 0.65239 | 1.5328 | -8.8684 |
| 25 | 0.21146 | 1904.4637 | -0.050421 | 0.76927 | 1.2999 | -11.79 |
| 26 | 0.1545 | 1690.5068 | -0.052253 | 0.81229 | 1.2311 | -11.1178 |
| 27 | 0.0066672 | 2338.8318 | -0.0099581 | 0.6753 | 1.4808 | -5.9407 |
| 28 | 0.45067 | 2133.3289 | -0.72879 | 0.62477 | 1.6006 | -17.4024 |
| 29 | 0.8281 | 1029.2922 | -0.40786 | 0.69237 | 1.4443 | -11.1277 |
| 30 | -0.6922 | 891.5253 | 0.04758 | 0.67053 | 1.4914 | -7.067 |
| 31 | -0.070821 | 1253.2769 | 0.051866 | 0.81753 | 1.2232 | -12.6865 |
| 32 | 0.23456 | 2132.0594 | 0.071736 | 0.85637 | 1.1677 | -24.1935 |
| 33 | 0.7841 | 824.6878 | -0.52374 | 0.79048 | 1.2651 | -11.2094 |
| 34 | -0.15907 | 1986.5752 | 0.38812 | 0.83835 | 1.1928 | -33.4541 |
| 35 | 0.15258 | 2681.409 | -0.18146 | 0.79497 | 1.2579 | -17.7585 |
| 36 | -0.67974 | 645.9023 | 0.13536 | 0.78638 | 1.2717 | -4.0444 |
| 37 | 0.55861 | 1117.1101 | -0.49198 | 0.76293 | 1.3107 | -21.9515 |
| 38 | -0.19884 | 949.6865 | 0.088635 | 0.69029 | 1.4487 | -6.3946 |
| 39 | 0.15089 | 3524.7826 | -0.41309 | 0.73746 | 1.356 | 4.2152 |

| | | | | | | |
|---|---|---|---|---|---|---|
| 40 | -0.20308 | 748.2264 | -0.86364 | 0.78305 | 1.2771 | 6.337 |
| 41 | -0.042758 | 1743.8754 | 0.20941 | 0.79027 | 1.2654 | -18.4403 |
| 42 | 0.61888 | 388.323 | -0.98428 | 0.67697 | 1.4772 | 1.026 |
| 43 | -0.2795 | 2832.5035 | 0.24458 | 0.81587 | 1.2257 | -18.5602 |
| 44 | -0.31005 | 2193.1529 | 0.30478 | 0.67194 | 1.4882 | -25.6332 |
| 45 | 0.23518 | 1764.6268 | 0.09949 | 0.73758 | 1.3558 | -11.6777 |
| 46 | 0.12831 | 902.702 | 0.031433 | 0.77845 | 1.2846 | -3.7735 |
| 47 | 0.23202 | 1888.417 | -0.17133 | 0.74917 | 1.3348 | -8.8833 |
| 48 | -0.086201 | 1553.3713 | 0.0082799 | 0.77389 | 1.2922 | -16.2786 |
| 49 | 0.15517 | 2807.0228 | -0.40672 | 0.57553 | 1.7375 | -38.0027 |
| 50 | -0.93308 | 1426.5465 | 0.47214 | 0.7264 | 1.3766 | -4.6457 |
| 51 | -0.096419 | 1520.0167 | -0.25702 | 0.73746 | 1.356 | -6.291 |
| 52 | -0.21517 | 2722.7512 | 0.10263 | 0.75581 | 1.3231 | -29.818 |
| 53 | -0.36436 | 3896.1541 | 0.20882 | 0.82123 | 1.2177 | -30.8803 |
| 54 | 0.68684 | 516.1792 | -0.29521 | 0.72603 | 1.3774 | -5.7547 |
| 55 | -0.042074 | 1857.854 | -0.192 | 0.78052 | 1.2812 | -9.529 |
| 56 | -0.54878 | 3998.5805 | 0.46845 | 0.67156 | 1.4891 | -13.1572 |
| 57 | -0.029825 | 3932.6764 | -0.065845 | 0.83137 | 1.2028 | -18.7982 |
| 58 | -0.43003 | 643.0891 | 0.55326 | 0.71252 | 1.4035 | -4.2515 |
| 59 | -0.14238 | 1030.0243 | 0.29374 | 0.80395 | 1.2439 | -10.6663 |
| 60 | 0.041156 | 2310.9615 | -0.050558 | 0.84182 | 1.1879 | -2.454 |
| 61 | -0.5936 | 2828.8926 | 0.68388 | 0.85513 | 1.1694 | -46.4409 |
| 62 | 0.28238 | 761.7348 | -0.53185 | 0.68811 | 1.4533 | -0.37912 |
| 63 | 0.14366 | 2244.2531 | -0.096128 | 0.73249 | 1.3652 | -37.0329 |
| 64 | -0.20054 | 3897.52 | 0.15015 | 0.76537 | 1.3066 | -6.0001 |
| 65 | 0.036683 | 1256.2988 | -0.046973 | 0.83504 | 1.1976 | -17.4838 |
| 66 | 1.2234 | 388.3378 | -0.98608 | 0.7624 | 1.3116 | -10.8104 |
| 67 | 0.61977 | 1574.0374 | -0.54646 | 0.69703 | 1.4347 | -8.3878 |
| 68 | -0.69033 | 1734.9401 | 0.48829 | 0.59438 | 1.6824 | -6.0776 |
| 69 | 0.11888 | 1716.155 | -0.12508 | 0.79356 | 1.2601 | -18.0172 |
| 70 | -0.76568 | 902.0037 | 0.37183 | 0.74701 | 1.3387 | -0.88596 |
| 71 | -0.039494 | 2242.8345 | 0.11961 | 0.6202 | 1.6124 | -20.2158 |
| 72 | 0.0067719 | 2138.3255 | -0.27652 | 0.77836 | 1.2848 | -6.0435 |
| 73 | -0.2588 | 951.7297 | 0.47599 | 0.76115 | 1.3138 | -8.803 |
| 74 | -1.0016 | 889.4405 | 1.8163 | 0.8089 | 1.2363 | -7.8651 |
| 75 | -0.40224 | 2397.4282 | 0.040045 | 0.7786 | 1.2843 | -16.0555 |
| 76 | 0.53815 | 1343.3724 | -0.19741 | 0.74067 | 1.3501 | -18.8057 |
| 77 | -0.072067 | 1874.3743 | 0.59616 | 0.82751 | 1.2084 | -37.2777 |
| 78 | -0.14767 | 2583.5642 | 0.33974 | 0.836 | 1.1962 | -28.5362 |
| 79 | 0.57607 | 3654.3071 | 0.092928 | 0.81787 | 1.2227 | -28.1191 |
| 80 | 0.59558 | 932.1737 | 1.4112 | 0.75481 | 1.3248 | -13.1087 |

| | | | | | | |
|---|---|---|---|---|---|---|
| 81 | 0.4088 | 1243.2203 | -0.40262 | 0.80824 | 1.2373 | -15.2995 |
| 82 | -0.3829 | 3404.2885 | 0.28156 | 0.86114 | 1.1613 | -40.0644 |
| 83 | 0.075764 | 4935.3441 | 0.45669 | 0.83419 | 1.1988 | -23.1148 |
| 84 | 0.28193 | 637.3764 | 0.073695 | 0.8284 | 1.2071 | -10.0203 |
| 85 | 0.52786 | 1236.6318 | -0.50093 | 0.75881 | 1.3179 | -27.8615 |
| 86 | -0.51385 | 2059.5799 | 0.024047 | 0.69465 | 1.4396 | -10.0982 |
| 87 | 0.22573 | 1386.4023 | 0.017432 | 0.7255 | 1.3784 | -25.6248 |
| 88 | -0.55143 | 3415.1697 | 0.43607 | 0.83348 | 1.1998 | -20.8188 |
| 89 | 0.21245 | 6721.0754 | 0.12343 | 0.89843 | 1.1131 | -30.5313 |
| 90 | 0.074465 | 1963.3446 | 0.1924 | 0.85886 | 1.1643 | -33.1776 |
| 91 | -1.3474 | 655.402 | 0.67662 | 0.66478 | 1.5043 | 0.769 |
| 92 | 0.18021 | 2603.9982 | 0.081053 | 0.69155 | 1.446 | -22.6147 |
| 93 | 0.29283 | 2376.3721 | -0.15157 | 0.76884 | 1.3007 | -30.8148 |
| 94 | -0.35036 | 2634.5243 | 0.005197 | 0.77653 | 1.2878 | -26.9254 |
| 95 | 0.25201 | 1344.9396 | -0.53766 | 0.80142 | 1.2478 | -19.1832 |
| 96 | 0.31675 | 461.3516 | 0.63856 | 0.67307 | 1.4857 | -3.2006 |
| 97 | -0.86109 | 1217.2528 | -0.05488 | 0.76819 | 1.3018 | -16.4403 |
| 98 | 0.090516 | 1757.3685 | 0.13608 | 0.77935 | 1.2831 | -28.4715 |
| 99 | 0.39593 | 1215.4315 | -0.49194 | 0.76985 | 1.299 | -12.8788 |
| 100 | 0.015991 | 2170.6092 | -0.14737 | 0.75453 | 1.3253 | -20.5929 |
| 101 | 0.32523 | 1422.5039 | 0.53729 | 0.76438 | 1.3082 | -9.0802 |
| 102 | 0.00078603 | 1904.8019 | 0.022086 | 0.76727 | 1.3033 | -28.737 |
| 103 | 0.32658 | 2178.6373 | -0.49359 | 0.72182 | 1.3854 | -21.3682 |
| 104 | 0.19014 | 2761.0426 | 0.41947 | 0.59406 | 1.6833 | -12.0935 |
| 105 | -0.14583 | 2915.4934 | -0.035473 | 0.55881 | 1.7895 | -10.1398 |
| 106 | 0.27238 | 2700.7792 | 0.27038 | 0.74069 | 1.3501 | -18.465 |
| 107 | 0.33945 | 890.983 | -0.066227 | 0.8008 | 1.2488 | -17.3253 |
| 108 | -0.17412 | 3825.0453 | 0.29083 | 0.80551 | 1.2415 | -30.4355 |
| 109 | 0.32326 | 972.7149 | -0.066742 | 0.80972 | 1.235 | -17.2802 |
| 110 | -0.091071 | 968.6202 | 0.074051 | 0.80118 | 1.2482 | -14.4725 |
| 111 | -0.64772 | 1649.6344 | 0.26961 | 0.67837 | 1.4741 | -22.2949 |
| 112 | 0.077216 | 2278.7042 | 0.46802 | 0.82306 | 1.215 | -28.4652 |

| Patient | Wavelet_HHL_stats_range_FB | LoG_sigma_3_mm_3D_glcm_clusProm_FB | Wavelet_HLH_glcm_correl1_FB | LoG_sigma_3_mm_2D_glcm_clusShade_FB | Wavelet_HLH_stats_min_FB | Wavelet_LLL_stats_max_FB |
|---|---|---|---|---|---|---|
| 1 | 72.5515 | 7051.3905 | 0.30696 | -217.1364 | -33.7231 | 72.4123 |
| 2 | 94.1545 | 1063.0069 | 0.26213 | -5.0763 | -34.3683 | 305.9109 |
| 3 | 74.0045 | 1510.2683 | 0.22628 | 7.9322 | -25.8659 | 167.8259 |
| 4 | 109.8245 | 829.8069 | 0.2274 | 12.3717 | -88.7166 | 1357.7351 |
| 5 | 140.5814 | 2568.4351 | 0.25143 | -32.6375 | -53.6967 | 227.3483 |
| 6 | 111.6469 | 1183.681 | 0.28129 | -16.0184 | -49.1173 | 245.1256 |
| 7 | 99.1869 | 615.4896 | 0.31113 | 16.1248 | -32.2618 | 322.5872 |
| 8 | 111.4955 | 4340.5382 | 0.28293 | -3.1113 | -44.4196 | 938.2278 |
| 9 | 115.051 | 332.6773 | 0.24417 | 13.6712 | -46.2831 | 619.029 |
| 10 | 102.8135 | 336.4637 | 0.12997 | -6.4838 | -103.7312 | 1352.7483 |
| 11 | 102.5267 | 1230.7145 | 0.27362 | -5.3672 | -52.4397 | 207.1107 |
| 12 | 78.4287 | 1203.1524 | 0.24667 | -12.5328 | -36.4498 | 348.1712 |
| 13 | 66.544 | 349.2557 | 0.24251 | 7.1001 | -37.0691 | 276.4866 |
| 14 | 103.6258 | 3174.1721 | 0.29633 | -45.0226 | -43.0625 | 412.8715 |
| 15 | 66.9022 | 799.6143 | 0.23616 | 8.8489 | -37.3733 | 179.2264 |
| 16 | 94.1515 | 2645.1808 | 0.27045 | 7.263 | -42.9854 | 169.6732 |
| 17 | 113.5243 | 1569.797 | 0.28567 | 0.98436 | -37.6285 | 432.4042 |
| 18 | 128.8009 | 16565.0966 | 0.29089 | -169.5963 | -61.924 | 657.9732 |
| 19 | 81.9325 | 131.0227 | 0.22786 | 2.8561 | -30.8341 | 302.3006 |
| 20 | 59.269 | 524.3533 | 0.27448 | 0.59088 | -41.0478 | 294.2781 |
| 21 | 83.6127 | 399.4885 | 0.24708 | -10.3463 | -31.4089 | 274.7357 |
| 22 | 98.9095 | 982.7564 | 0.2326 | -4.6636 | -57.5326 | 354.4508 |
| 23 | 106.6227 | 468.6728 | 0.25048 | 16.4899 | -44.9678 | 516.2502 |
| 24 | 108.7292 | 5904.1881 | 0.27042 | -111.4184 | -38.2376 | 224.7796 |
| 25 | 80.4857 | 872.1726 | 0.2684 | 9.3842 | -40.2451 | 373.7802 |
| 26 | 117.7012 | 793.1117 | 0.2266 | 8.0296 | -35.8917 | 411.8757 |
| 27 | 113.7879 | 2249.5948 | 0.27094 | -4.1804 | -58.6824 | 370.4321 |
| 28 | 79.5399 | 1501.4787 | 0.28791 | 40.348 | -41.4978 | 360.2708 |
| 29 | 95.2195 | 564.3519 | 0.27757 | 20.7624 | -52.1683 | 321.0289 |
| 30 | 104.1634 | 2447.098 | 0.23297 | -45.417 | -57.6731 | 352.77 |
| 31 | 84.3815 | 673.9146 | 0.2531 | 5.8193 | -43.6501 | 169.2921 |
| 32 | 56.0846 | 1025.646 | 0.26798 | 8.3208 | -23.6415 | 142.4491 |
| 33 | 67.5209 | 372.106 | 0.23161 | 22.9338 | -52.2843 | 191.7209 |
| 34 | 71.7028 | 2028.6893 | 0.31301 | -11.6724 | -30.5452 | 124.7866 |
| 35 | 67.504 | 941.1981 | 0.31519 | 10.7052 | -49.9711 | 255.684 |
| 36 | 63.6785 | 195.3849 | 0.25128 | -5.5145 | -35.5322 | 160.6152 |
| 37 | 112.149 | 1195.6584 | 0.23957 | 42.5137 | -42.2653 | 165.9093 |
| 38 | 65.8625 | 330.7922 | 0.25387 | -1.5708 | -28.9941 | 312.7067 |
| 39 | 176.2654 | 6471.4307 | 0.2751 | 39.9599 | -72.9584 | 361.0067 |

| | | | | | | |
|---|---|---|---|---|---|---|
| 40 | 66.9197 | 1175.6272 | 0.28362 | 0.32617 | -40.645 | 318.8042 |
| 41 | 65.6662 | 643.9789 | 0.2623 | 1.1126 | -41.1709 | 167.1869 |
| 42 | 53.7527 | 156.814 | 0.21406 | 6.0216 | -57.6718 | 521.4413 |
| 43 | 119.5832 | 1864.9513 | 0.25562 | -17.115 | -37.7526 | 141.3369 |
| 44 | 99.2 | 1440.7628 | 0.29297 | -13.8775 | -35.224 | 221.3228 |
| 45 | 104.3989 | 1616.0944 | 0.26033 | 24.7603 | -41.2724 | 213.9988 |
| 46 | 61.5387 | 184.2801 | 0.2442 | 2.3625 | -26.7888 | 179.8807 |
| 47 | 76.7769 | 515.8877 | 0.27499 | 8.1856 | -40.118 | 195.4081 |
| 48 | 99.1275 | 2007.2987 | 0.25952 | 9.7016 | -38.1078 | 286.3909 |
| 49 | 113.0496 | 8539.1572 | 0.2803 | 21.3268 | -41.5048 | 251.9802 |
| 50 | 97.5826 | 2502.5519 | 0.22731 | -55.9981 | -55.7228 | 263.8318 |
| 51 | 111.8893 | 1262.8819 | 0.25864 | 2.9696 | -39.8188 | 304.6579 |
| 52 | 73.537 | 2331.7349 | 0.30264 | -23.4979 | -57.2459 | 13.2085 |
| 53 | 61.0737 | 1577.7804 | 0.25466 | -20.5292 | -38.2014 | 104.9768 |
| 54 | 95.0035 | 184.9338 | 0.22192 | 8.22 | -28.197 | 396.6098 |
| 55 | 63.6039 | 782.7032 | 0.26134 | 1.1431 | -38.7952 | 293.9885 |
| 56 | 142.7237 | 5100.3609 | 0.28286 | -60.2315 | -56.2768 | 372.4091 |
| 57 | 76.3681 | 1981.7118 | 0.29802 | -4.2008 | -25.9741 | 117.9854 |
| 58 | 80.8013 | 271.1645 | 0.21136 | -4.0259 | -36.1637 | 352.5338 |
| 59 | 98.6895 | 746.3473 | 0.26931 | 5.3733 | -46.6533 | 237.0342 |
| 60 | 86.7955 | 1133.333 | 0.22005 | 3.8417 | -29.373 | 143.1915 |
| 61 | 61.4213 | 1564.5989 | 0.30867 | -38.7748 | -29.4476 | 362.4428 |
| 62 | 85.4318 | 890.2207 | 0.23304 | 11.5813 | -34.3771 | -83.8225 |
| 63 | 73.1348 | 1782.6076 | 0.29144 | 30.4729 | -44.0575 | 191.2674 |
| 64 | 65.6003 | 3829.9649 | 0.29829 | -37.2437 | -38.3643 | 296.5485 |
| 65 | 60.7692 | 1029.3916 | 0.25687 | 0.01108 | -27.1396 | 90.1546 |
| 66 | 72.6651 | 177.2534 | 0.21315 | 15.6805 | -54.5803 | 352.1513 |
| 67 | 116.9804 | 573.5873 | 0.28214 | 20.8671 | -48.9026 | 250.2455 |
| 68 | 111.4256 | 3690.7804 | 0.22747 | -66.9242 | -62.8215 | 223.8533 |
| 69 | 89.3352 | 641.2977 | 0.26313 | 9.9947 | -41.8407 | 260.5807 |
| 70 | 75.7392 | 808.553 | 0.27691 | -20.4965 | -68.4415 | 402.2644 |
| 71 | 93.0372 | 1754.5132 | 0.25799 | -8.5124 | -31.6148 | 158.2015 |
| 72 | 111.7844 | 1479.2185 | 0.28721 | -3.366 | -36.7679 | 128.0775 |
| 73 | 89.4235 | 873.4321 | 0.26345 | -6.3263 | -38.1972 | 259.1891 |
| 74 | 109.6509 | 2235.9165 | 0.2893 | -48.4873 | -57.6467 | 620.1388 |
| 75 | 106.0898 | 2107.6932 | 0.28042 | -21.4627 | -81.1792 | 390.6434 |
| 76 | 102.5225 | 964.8676 | 0.24192 | 30.6018 | -57.431 | 265.9828 |
| 77 | 128.4303 | 1329.6293 | 0.24817 | 10.5716 | -43.3493 | 197.1496 |
| 78 | 89.2489 | 2921.0112 | 0.29027 | -20.9162 | -31.4685 | 167.114 |
| 79 | 76.89 | 2209.9394 | 0.27702 | 53.3933 | -44.7508 | 140.3049 |
| 80 | 118.9611 | 316.0501 | 0.21478 | 13.1846 | -28.1552 | 234.6016 |

| | | | | | | |
|---|---|---|---|---|---|---|
| 81 | 57.7169 | 277.9353 | 0.22943 | 6.5542 | -26.9896 | 223.1065 |
| 82 | 107.7536 | 1148.2052 | 0.28517 | -25.4299 | -24.0504 | 195.1325 |
| 83 | 98.842 | 2336.8379 | 0.28574 | 17.2828 | -41.4103 | 365.0439 |
| 84 | 58.1942 | 121.8111 | 0.23217 | 5.9821 | -45.0789 | 122.8023 |
| 85 | 80.3444 | 644.0518 | 0.28406 | 29.8477 | -50.7494 | 184.2535 |
| 86 | 120.535 | 8208.7839 | 0.27082 | -98.5425 | -58.9281 | 337.2505 |
| 87 | 98.3177 | 1260.2543 | 0.23977 | 29.8402 | -58.1276 | 303.7733 |
| 88 | 85.0066 | 4068.835 | 0.26914 | -95.4186 | -45.4008 | 241.2818 |
| 89 | 66.2496 | 1470.1602 | 0.18639 | -6.2148 | -22.3359 | -61.0974 |
| 90 | 53.2649 | 602.152 | 0.33056 | 3.3517 | -45.3366 | 172.6321 |
| 91 | 113.6847 | 743.5611 | 0.20155 | -23.6732 | -61.4037 | 984.8512 |
| 92 | 88.2183 | 1835.4392 | 0.26966 | 29.717 | -34.4382 | 201.9343 |
| 93 | 85.6329 | 864.6791 | 0.27915 | 16.8982 | -53.0144 | 200.4765 |
| 94 | 89.43 | 2992.7439 | 0.25038 | -61.6495 | -51.2142 | -8.0089 |
| 95 | 89.0944 | 718.0889 | 0.26746 | 16.9952 | -35.4178 | 228.4104 |
| 96 | 111.2783 | 204.1864 | 0.20154 | 3.081 | -45.6024 | 566.7353 |
| 97 | 62.0707 | 1189.0419 | 0.22799 | -28.0694 | -47.4151 | 365.4718 |
| 98 | 78.5929 | 749.3342 | 0.26321 | 9.2575 | -27.401 | 394.5742 |
| 99 | 82.0459 | 832.7156 | 0.24979 | 24.7953 | -37.1004 | 296.2988 |
| 100 | 77.5061 | 908.2061 | 0.24709 | 0.22129 | -26.4044 | 91.4708 |
| 101 | 65.1464 | 578.3533 | 0.2558 | 8.5646 | -35.7998 | 217.6161 |
| 102 | 75.1964 | 1574.1172 | 0.25936 | 4.3691 | -39.4454 | 64.5462 |
| 103 | 71.6024 | 973.1526 | 0.27215 | 17.6026 | -42.9093 | 326.5725 |
| 104 | 120.2247 | 679.0298 | 0.28541 | 10.1633 | -56.9908 | 208.4469 |
| 105 | 146.6453 | 4616.0789 | 0.26663 | -29.0411 | -70.1469 | 371.8825 |
| 106 | 87.804 | 884.7841 | 0.27062 | 19.5664 | -47.21 | 249.55 |
| 107 | 68.2124 | 565.8208 | 0.24822 | 10.5239 | -37.1296 | 400.7409 |
| 108 | 116.1178 | 2238.9417 | 0.23042 | -8.9921 | -41.3957 | 169.0003 |
| 109 | 60.2888 | 432.5573 | 0.26362 | 15.1043 | -29.2154 | 145.755 |
| 110 | 81.8819 | 506.5145 | 0.28378 | -5.8535 | -39.8476 | -549.3492 |
| 111 | 126.7801 | 2735.6072 | 0.23425 | -47.0685 | -61.7431 | 424.391 |
| 112 | 94.2814 | 478.9221 | 0.28589 | 10.4266 | -26.2048 | 141.6223 |

| Patient | Wavelet_HLL_stats_max_FB | LoG_sigma_3_mm_3D_glcm_homogeneity1_AIP | Wavelet_HHL_stats_range_AIP | GLCM_correl1_AIP | Wavelet_LHH_glszm_largeAreaEmphasis_AIP | Wavelet_LLL_glcm_clusShade_AIP |
|---|---|---|---|---|---|---|
| 1 | 92.3554 | 0.48431 | 54.1571 | 0.70441 | 171.5217 | 415.7152 |
| 2 | 159.8651 | 0.65469 | 39.5987 | 0.89782 | 239.6471 | 653.2389 |
| 3 | 172.0044 | 0.65968 | 38.676 | 0.84686 | 2680.2727 | 17.3788 |
| 4 | 214.185 | 0.70335 | 110.1923 | 0.82617 | 6516574.667 | -1881.0636 |
| 5 | 199.4582 | 0.65579 | 123.1914 | 0.83865 | 549990.3684 | -2157.4014 |
| 6 | 231.8443 | 0.68642 | 49.8807 | 0.94398 | 256197.6176 | -1892.266 |
| 7 | 180.2212 | 0.62991 | 63.3736 | 0.87981 | 6288.6429 | -3804.3826 |
| 8 | 191.713 | 0.49232 | 168.0715 | 0.71412 | 91.25 | -3085.0241 |
| 9 | 181.3438 | 0.74503 | 183.2954 | 0.83597 | 56327855 | -1469.1052 |
| 10 | 239.9834 | 0.75923 | 84.1523 | 0.80898 | 3445859 | -228.2117 |
| 11 | 167.3623 | 0.64871 | 36.4602 | 0.9004 | 92935.4667 | 357.7479 |
| 12 | 148.8832 | 0.59389 | 34.1524 | 0.88143 | 9944.5357 | -22.4844 |
| 13 | 157.0229 | 0.64873 | 116.3817 | 0.7586 | 413314.9333 | -858.1668 |
| 14 | 200.0578 | 0.5975 | 75.8534 | 0.84521 | 108286.0833 | -4535.6752 |
| 15 | 142.6737 | 0.55408 | 66.9279 | 0.72829 | 17718.5517 | -1122.131 |
| 16 | 216.7343 | 0.61392 | 58.5521 | 0.90326 | 5049.8333 | 1016.674 |
| 17 | 212.8955 | 0.56846 | 69.7718 | 0.86459 | 17201.8065 | -904.2355 |
| 18 | 212.0837 | 0.44763 | 104.2523 | 0.81057 | 5.2889 | -7277.5186 |
| 19 | 202.8186 | 0.75244 | 40.2841 | 0.80527 | 446049.9583 | -66.2645 |
| 20 | 126.5483 | 0.66447 | 29.0461 | 0.90204 | 16185.5556 | 703.2986 |
| 21 | 113.9562 | 0.68239 | 59.7481 | 0.92309 | 147122.3077 | -367.0443 |
| 22 | 142.5991 | 0.63328 | 45.929 | 0.85499 | 68902.6786 | -1009.7565 |
| 23 | 146.9346 | 0.65012 | 101.2388 | 0.78204 | 4.575 | -1356.2813 |
| 24 | 257.14 | 0.60566 | 37.2404 | 0.91114 | 14126.7813 | -883.0377 |
| 25 | 156.8409 | 0.66399 | 57.7496 | 0.87539 | 492283.9706 | -2261.3555 |
| 26 | 140.0386 | 0.64783 | 55.2845 | 0.89532 | 35533.9688 | -1131.1406 |
| 27 | 193.8843 | 0.56486 | 77.4457 | 0.90457 | 84242.2051 | 317.2208 |
| 28 | 147.7568 | 0.59848 | 54.1681 | 0.79371 | 1136.3714 | -1853.7148 |
| 29 | 201.0824 | 0.72868 | 54.7354 | 0.95323 | 10269855.03 | -3761.0253 |
| 30 | 213.1548 | 0.64872 | 46.5384 | 0.89331 | 21.4483 | -1016.5618 |
| 31 | 214.5397 | 0.65806 | 67.8079 | 0.8689 | 25797.5 | -1542.8514 |
| 32 | 134.3368 | 0.67628 | 32.2597 | 0.90198 | 3763.5926 | 165.8203 |
| 33 | 175.7911 | 0.70439 | 40.9924 | 0.90526 | 288458.2647 | -2455.0516 |
| 34 | 132.4292 | 0.59487 | 47.4632 | 0.86507 | 17.1714 | -370.5353 |
| 35 | 154.905 | 0.52333 | 68.2554 | 0.80926 | 57442.303 | -3066.2283 |
| 36 | 125.031 | 0.68619 | 65.9179 | 0.77814 | 170231 | -515.1736 |
| 37 | 155.7783 | 0.58631 | 101.9278 | 0.78783 | 12.1471 | -2509.8053 |
| 38 | 129.9754 | 0.66705 | 72.5361 | 0.83708 | 67268.7429 | -1015.239 |
| 39 | 254.2047 | 0.51062 | 98.508 | 0.91989 | 6.8 | 3447.7452 |

| | | | | | | |
|---|---|---|---|---|---|---|
| 40 | 82.7541 | 0.65686 | 29.3384 | 0.90216 | 52068.3043 | -115.863 |
| 41 | 152.7761 | 0.64333 | 40.2985 | 0.88143 | 9032 | -615.8618 |
| 42 | 94.6104 | 0.75249 | 34.5291 | 0.96811 | 2622581.033 | -1712.5698 |
| 43 | 155.2288 | 0.56426 | 62.8237 | 0.83676 | 202.3667 | 69.8304 |
| 44 | 156.1288 | 0.52499 | 72.4297 | 0.78263 | 38.7857 | -1517.7669 |
| 45 | 215.197 | 0.6173 | 113.444 | 0.85611 | 286540.4615 | -4741.5825 |
| 46 | 114.0949 | 0.59115 | 54.8972 | 0.7834 | 8694.3043 | -342.3278 |
| 47 | 205.1388 | 0.65927 | 68.2972 | 0.85359 | 2.6471 | -2077.3771 |
| 48 | 162.3721 | 0.639 | 47.2475 | 0.92277 | 49568.8 | 501.8615 |
| 49 | 131.1174 | 0.43441 | 116.2441 | 0.84176 | 24.619 | 2604.2254 |
| 50 | 229.3103 | 0.68211 | 45.6383 | 0.94251 | 58203.2581 | -642.7875 |
| 51 | 203.6599 | 0.64215 | 104.5713 | 0.90146 | 924105.7568 | -1920.1056 |
| 52 | 150.3982 | 0.46587 | 54.2508 | 0.69956 | 4.5161 | -512.2985 |
| 53 | 195.6364 | 0.45874 | 74.8003 | 0.69459 | 198.5625 | -435.0152 |
| 54 | 154.8898 | 0.76431 | 54.5751 | 0.96524 | 8786145.464 | -1851.4331 |
| 55 | 122.6354 | 0.67659 | 43.0639 | 0.90984 | 43936.6 | 503.0393 |
| 56 | 281.9961 | 0.5751 | 104.0683 | 0.87005 | 280891.8182 | -3068.2502 |
| 57 | 95.4819 | 0.48262 | 63.7117 | 0.78634 | 353.2857 | -404.4843 |
| 58 | 163.6425 | 0.73582 | 35.8674 | 0.92363 | 181870.92 | -339.6446 |
| 59 | 174.2463 | 0.65125 | 67.948 | 0.8427 | 100692.5357 | -1429.6337 |
| 60 | 119.1498 | 0.59848 | 30.1119 | 0.87562 | 6489.6563 | -587.1304 |
| 61 | 146.4541 | 0.50132 | 31.4308 | 0.72237 | 13.375 | -154.6385 |
| 62 | 141.127 | 0.59396 | 50.4712 | 0.87978 | 487701.88 | -66.3609 |
| 63 | 108.8714 | 0.49729 | 59.3819 | 0.73877 | 2326.2667 | -964.0768 |
| 64 | 138.7286 | 0.49904 | 66.1551 | 0.84321 | 6.0857 | -908.9024 |
| 65 | 99.5042 | 0.55928 | 40.2149 | 0.81943 | 960.7778 | 414.2229 |
| 66 | 121.7526 | 0.79971 | 40.1958 | 0.77326 | 10889837.24 | -70.1927 |
| 67 | 169.8631 | 0.70899 | 56.6697 | 0.85409 | 248844.2857 | -2027.1367 |
| 68 | 206.6098 | 0.57954 | 93.5135 | 0.86447 | 36.6296 | -451.836 |
| 69 | 153.1533 | 0.62175 | 38.064 | 0.81013 | 37483.3704 | -802.7963 |
| 70 | 176.5748 | 0.67609 | 36.9464 | 0.93369 | 518144.9259 | 96.509 |
| 71 | 118.7827 | 0.47138 | 104.5816 | 0.78381 | 31.3548 | 388.4632 |
| 72 | 283.7155 | 0.68025 | 39.5467 | 0.93012 | 51137.6207 | 668.9658 |
| 73 | 185.4219 | 0.65444 | 68.328 | 0.87535 | 138759.9063 | -2277.8642 |
| 74 | 224.0459 | 0.72048 | 126.7329 | 0.94551 | 20024727.61 | -1956.9732 |
| 75 | 194.5066 | 0.59776 | 57.2188 | 0.87114 | 9610.5 | -418.9557 |
| 76 | 195.3369 | 0.65172 | 51.0102 | 0.82089 | 12712.75 | -764.0955 |
| 77 | 104.7346 | 0.48172 | 86.9791 | 0.7313 | 19.8667 | -1101.9908 |
| 78 | 122.9628 | 0.50215 | 58.3803 | 0.74158 | 29.5926 | -412.4289 |
| 79 | 193.2861 | 0.53761 | 59.494 | 0.81452 | 14.1818 | -1888.353 |
| 80 | 139.2569 | 0.71205 | 69.989 | 0.84934 | 132947.52 | -849.6022 |

| | | | | | | |
|---|---|---|---|---|---|---|
| 81 | 121.4365 | 0.70513 | 38.2355 | 0.77023 | 9390.0952 | -160.312 |
| 82 | 163.1432 | 0.40714 | 70.299 | 0.68602 | 227.2667 | -2294.6661 |
| 83 | 249.0093 | 0.57124 | 45.6735 | 0.82544 | 664.9167 | 56.2349 |
| 84 | 115.8078 | 0.72184 | 33.2123 | 0.72894 | 36120.0556 | -112.137 |
| 85 | 140.4146 | 0.60484 | 111.4388 | 0.69461 | 47845.8235 | -980.6016 |
| 86 | 294.4683 | 0.52199 | 118.3109 | 0.81335 | 427545.5417 | -7490.8882 |
| 87 | 200.7649 | 0.5484 | 89.0817 | 0.80686 | 4272.2059 | -2199.6462 |
| 88 | 170.8328 | 0.60242 | 49.3263 | 0.82882 | 4255.037 | -4.7678 |
| 89 | 167.2945 | 0.51815 | 54.4497 | 0.69917 | 8.4815 | 187.7027 |
| 90 | 93.468 | 0.59329 | 44.2129 | 0.82513 | 468.7407 | -616.5373 |
| 91 | 226.6982 | 0.68677 | 107.004 | 0.94434 | 17188875.84 | -909.2152 |
| 92 | 184.1217 | 0.53951 | 50.1258 | 0.8243 | 12.2 | -1234.0997 |
| 93 | 159.623 | 0.56119 | 58.5503 | 0.82556 | 3.2692 | -371.5433 |
| 94 | 114.8526 | 0.46188 | 60.4415 | 0.65144 | 81.625 | -337.1588 |
| 95 | 118.5966 | 0.66107 | 34.5359 | 0.88954 | 6260.4063 | -153.8322 |
| 96 | 170.991 | 0.73794 | 67.2669 | 0.91575 | 4294015.276 | -628.851 |
| 97 | 145.0703 | 0.7097 | 37.7986 | 0.8531 | 72272.0909 | -139.8633 |
| 98 | 158.658 | 0.60016 | 46.0516 | 0.80932 | 2666.7037 | -495.3779 |
| 99 | 135.0012 | 0.59634 | 82.1584 | 0.72494 | 23892.8387 | -1180.0419 |
| 100 | 123.5325 | 0.50058 | 59.9024 | 0.69759 | 257.1667 | 103.8212 |
| 101 | 144.098 | 0.72043 | 32.3108 | 0.94751 | 130207.7667 | -839.5755 |
| 102 | 150.3473 | 0.45785 | 100.2435 | 0.81521 | 19.5152 | -393.8395 |
| 103 | 147.202 | 0.66161 | 32.768 | 0.88367 | 1806.0333 | -322.3525 |
| 104 | 292.9915 | 0.62187 | 67.4979 | 0.76052 | 128156.7037 | -938.8145 |
| 105 | 242.1818 | 0.48865 | 152.6672 | 0.87594 | 3.4872 | 5.8521 |
| 106 | 123.9017 | 0.5094 | 73.6239 | 0.76851 | 5.3333 | -1863.3785 |
| 107 | 111.2709 | 0.66722 | 54.1387 | 0.92523 | 30975.2121 | -972.1638 |
| 108 | 140.7376 | 0.45224 | 112.1822 | 0.69022 | 156.9091 | -2439.0078 |
| 109 | 129.439 | 0.57896 | 88.2457 | 0.65969 | 4209.2759 | -394.8184 |
| 110 | 79.308 | 0.51203 | 95.8519 | 0.7069 | 1651.6364 | -47.8925 |
| 111 | 164.9898 | 0.56482 | 105.0619 | 0.78684 | 6353.7931 | -919.2539 |
| 112 | 143.1821 | 0.52586 | 91.4176 | 0.68414 | 1693.9655 | -873.124 |

| Patient | LoG_sigma_3_mm_3D_stats_skewness_AIP | Wavelet_LLH_stats_skewness_AIP | Shape_spherDisprop_AIP | LoG_sigma_3_mm_2D_glcm_clusProm_AIP | LoG_sigma_3_mm_2D_stats_skewness_AIP | Wavelet_HHL_stats_kurtosis_AIP | Shape_sphericity_AIP |
|---|---|---|---|---|---|---|---|
| 1 | 0.19813 | 0.10597 | 1.4537 | 392.846 | 0.20965 | 3.3606 | 0.68788 |
| 2 | -0.092863 | 0.055561 | 1.2321 | 56.197 | -0.071329 | 5.7026 | 0.81165 |
| 3 | 0.037662 | 1.9892 | 1.3542 | 88.8496 | -0.087068 | 4.7329 | 0.73844 |
| 4 | 0.061299 | -0.11937 | 1.3642 | 382.0844 | -0.063852 | 13.4088 | 0.73303 |
| 5 | -0.47258 | 0.10855 | 1.7295 | 849.399 | -0.29951 | 9.1365 | 0.5782 |
| 6 | -0.40784 | 0.65785 | 1.2973 | 99.0602 | -0.4709 | 7.0283 | 0.77082 |
| 7 | -0.052647 | -0.16325 | 1.5738 | 155.6327 | -0.17074 | 5.5708 | 0.6354 |
| 8 | 0.36665 | -0.019182 | 1.7341 | 2757.8181 | 0.40612 | 7.685 | 0.57666 |
| 9 | 0.73024 | -0.72234 | 1.4105 | 297.6917 | 0.71066 | 28.2557 | 0.70895 |
| 10 | 0.52886 | 4.0336 | 1.5464 | 62.4443 | -0.18036 | 17.8156 | 0.64665 |
| 11 | 0.029521 | 0.065628 | 1.3718 | 110.1761 | -1.035 | 5.7239 | 0.72895 |
| 12 | 0.11078 | 0.38817 | 1.4325 | 214.2261 | 0.11504 | 4.7742 | 0.69806 |
| 13 | 0.18863 | -0.056262 | 1.5515 | 307.6558 | 0.041848 | 9.6786 | 0.64453 |
| 14 | -0.43665 | 0.42339 | 1.481 | 852.4135 | -0.5827 | 6.1461 | 0.6752 |
| 15 | -0.17298 | 0.22308 | 1.2936 | 661.9525 | -0.040417 | 5.2002 | 0.77306 |
| 16 | 0.26742 | -0.087043 | 1.378 | 129.2558 | 0.30486 | 5.0496 | 0.72569 |
| 17 | -0.19906 | 0.046163 | 1.4533 | 337.6735 | -0.12585 | 6.1222 | 0.6881 |
| 18 | -0.8936 | 0.026557 | 1.5803 | 10797.1561 | -0.66814 | 6.0508 | 0.63278 |
| 19 | 0.088646 | 0.13005 | 1.3681 | 21.7862 | 0.16618 | 7.219 | 0.73092 |
| 20 | 0.70384 | -0.23034 | 1.2102 | 23.9664 | 0.19602 | 4.803 | 0.82629 |
| 21 | -0.12719 | 0.28228 | 1.4905 | 44.3878 | -0.27036 | 8.2927 | 0.67089 |
| 22 | -0.40763 | 0.31821 | 1.4709 | 133.9964 | -0.36649 | 4.9885 | 0.67986 |
| 23 | 0.39183 | -0.50134 | 1.4619 | 345.4702 | 0.55877 | 10.4134 | 0.68404 |
| 24 | -0.86999 | 2.3091 | 1.5255 | 516.5818 | -0.98149 | 5.1443 | 0.65552 |
| 25 | 0.08567 | -0.07886 | 1.4425 | 183.4978 | 0.27939 | 8.3885 | 0.69322 |
| 26 | -0.19784 | 0.23476 | 1.3668 | 106.5138 | -0.21023 | 6.2932 | 0.73161 |
| 27 | -0.11285 | 0.07206 | 1.5247 | 372.1742 | -0.18339 | 5.9537 | 0.65587 |
| 28 | 0.010721 | -0.15724 | 1.4227 | 343.9132 | 0.40028 | 5.8946 | 0.70289 |
| 29 | 0.43158 | -0.33086 | 1.3999 | 57.067 | 0.42824 | 11.6685 | 0.71436 |
| 30 | -0.51185 | 0.1266 | 1.6636 | 225.1469 | -0.44271 | 5.7293 | 0.60109 |
| 31 | -0.31803 | 0.58606 | 1.2707 | 138.8103 | -0.14946 | 7.2349 | 0.78696 |
| 32 | 0.49086 | -0.23865 | 1.2595 | 32.1919 | 0.22991 | 5.3975 | 0.79396 |
| 33 | 0.35429 | -0.40149 | 1.2756 | 120.4598 | 0.54004 | 7.2326 | 0.78397 |
| 34 | 0.079923 | 0.74249 | 1.2414 | 150.7714 | 0.019365 | 5.5065 | 0.80552 |
| 35 | -0.024273 | -0.031012 | 1.3367 | 618.3694 | 0.1023 | 5.6674 | 0.74812 |
| 36 | -0.79757 | 0.78669 | 1.3577 | 135.2074 | -0.64348 | 6.9652 | 0.73656 |
| 37 | 0.12058 | -0.11358 | 1.4017 | 720.5642 | 0.40968 | 7.7714 | 0.71343 |
| 38 | 0.18979 | -0.017449 | 1.501 | 110.148 | 0.046634 | 6.8366 | 0.66623 |
| 39 | 0.02096 | 0.68229 | 1.4767 | 2478.0927 | -0.16527 | 6.5081 | 0.6772 |

| | | | | | | | |
|---|---|---|---|---|---|---|---|
| 40 | -1.2544 | 2.826 | 1.3601 | 457.8914 | -1.6927 | 4.8215 | 0.73526 |
| 41 | -0.12355 | -0.025295 | 1.3206 | 47.3304 | -0.080832 | 5.0149 | 0.75724 |
| 42 | 0.42366 | -0.10629 | 1.7773 | 22.8939 | 0.58701 | 6.6822 | 0.56265 |
| 43 | -0.2798 | -0.20219 | 1.2908 | 287.9275 | -0.16063 | 7.0929 | 0.7747 |
| 44 | -0.26526 | 0.3047 | 1.5164 | 641.9598 | -0.3302 | 5.1218 | 0.65945 |
| 45 | 0.16922 | -0.472 | 1.4456 | 781.1383 | 0.2032 | 7.945 | 0.69177 |
| 46 | -0.38332 | 0.34317 | 1.3117 | 209.0066 | -0.16245 | 5.1336 | 0.76236 |
| 47 | 0.16611 | -0.15856 | 1.3715 | 137.2385 | 0.3098 | 7.3771 | 0.72914 |
| 48 | 0.21937 | -0.29765 | 1.436 | 176.8419 | 0.27555 | 5.3119 | 0.6964 |
| 49 | 0.34916 | -0.29311 | 1.5666 | 2877.8209 | 0.23662 | 5.5241 | 0.63833 |
| 50 | -0.68937 | 0.081726 | 1.3957 | 115.4471 | -0.71562 | 5.7782 | 0.7165 |
| 51 | 0.10108 | 0.097094 | 1.4649 | 244.4665 | 0.057605 | 10.2077 | 0.68264 |
| 52 | -0.26422 | 0.58617 | 1.3219 | 1074.9068 | -0.3336 | 4.0329 | 0.75649 |
| 53 | -0.33893 | 0.41814 | 1.1974 | 553.8529 | -0.42845 | 4.3891 | 0.83515 |
| 54 | 0.40618 | -0.53107 | 1.4786 | 17.1777 | 0.22128 | 10.6561 | 0.67633 |
| 55 | -0.08154 | -0.16375 | 1.4047 | 37.5983 | -0.035076 | 7.0797 | 0.71192 |
| 56 | -0.71387 | -0.05701 | 1.5186 | 636.3929 | -0.53933 | 6.7657 | 0.65851 |
| 57 | -0.12308 | 0.17684 | 1.2979 | 428.6995 | 0.14381 | 5.5637 | 0.77049 |
| 58 | -0.4511 | 0.7869 | 1.3866 | 26.7082 | -0.44754 | 5.5686 | 0.72117 |
| 59 | 0.078625 | 0.16054 | 1.3411 | 119.4973 | 0.18884 | 7.6318 | 0.74568 |
| 60 | 0.19437 | 1.0127 | 1.2127 | 310.1155 | 0.079795 | 4.0826 | 0.82459 |
| 61 | -0.28602 | 0.33966 | 1.2193 | 545.5773 | -0.37554 | 3.8381 | 0.82017 |
| 62 | -0.07028 | 1.5457 | 1.492 | 310.0009 | -0.18401 | 5.3429 | 0.67026 |
| 63 | 0.08638 | 0.23777 | 1.2777 | 1482.3554 | 0.23856 | 5.1495 | 0.78267 |
| 64 | -0.082203 | -0.3112 | 1.3401 | 525.1709 | -0.262 | 4.3729 | 0.74619 |
| 65 | 0.061434 | 0.48201 | 1.2912 | 159.7409 | 0.050615 | 3.82 | 0.77445 |
| 66 | 1.0184 | -0.86956 | 1.4196 | 16.521 | 1.1641 | 12.7703 | 0.7044 |
| 67 | 0.50943 | -0.14028 | 1.4611 | 81.2885 | 0.46145 | 10.0371 | 0.6844 |
| 68 | -0.062933 | 0.069958 | 1.6763 | 468.0122 | -0.15908 | 7.7328 | 0.59656 |
| 69 | -0.067868 | 0.1337 | 1.3151 | 165.6199 | -0.11436 | 5.6886 | 0.76038 |
| 70 | -0.89563 | 2.7099 | 1.3692 | 188.9673 | -1.3676 | 6.3023 | 0.73033 |
| 71 | -0.11414 | -0.35655 | 1.6938 | 922.6429 | -0.0006656 | 4.377 | 0.59038 |
| 72 | 0.24498 | -0.3006 | 1.2896 | 42.0312 | 0.28701 | 5.1311 | 0.77542 |
| 73 | -0.52202 | 0.45166 | 1.3265 | 201.5486 | -0.4803 | 7.6465 | 0.75389 |
| 74 | -1.344 | 0.6508 | 1.3884 | 361.9355 | -1.8286 | 69.4464 | 0.72027 |
| 75 | -0.45668 | 0.0076797 | 1.3805 | 251.7687 | -0.40691 | 5.4483 | 0.72438 |
| 76 | -0.048424 | -0.33651 | 1.2717 | 138.6181 | 0.10056 | 6.1141 | 0.78634 |
| 77 | -0.11187 | 0.312 | 1.2306 | 542.2844 | 0.03075 | 6.9144 | 0.81264 |
| 78 | -0.18094 | -0.164 | 1.2057 | 427.4933 | -0.20538 | 3.9415 | 0.8294 |
| 79 | -0.19824 | 0.12333 | 1.2335 | 401.4238 | 0.13162 | 5.6508 | 0.81069 |
| 80 | 0.15751 | -0.10671 | 1.2847 | 81.6462 | 0.033623 | 11.3187 | 0.77838 |

| | | | | | | | |
|---|---|---|---|---|---|---|---|
| 81 | 0.077447 | 0.77378 | 1.1745 | 21.0923 | 0.090357 | 6.1157 | 0.85142 |
| 82 | -0.54275 | 0.99543 | 1.2189 | 874.0648 | -0.41892 | 3.021 | 0.82038 |
| 83 | 0.22394 | -0.13361 | 1.2502 | 232.5881 | 0.28568 | 4.6748 | 0.79988 |
| 84 | -0.22787 | 0.41603 | 1.2555 | 28.0665 | -0.1375 | 5.319 | 0.7965 |
| 85 | 0.24851 | -0.39653 | 1.2825 | 450.0851 | 0.36255 | 10.9628 | 0.77974 |
| 86 | -1.0182 | 0.39237 | 1.6016 | 5737.0481 | -0.54036 | 6.9339 | 0.62437 |
| 87 | -0.13018 | -0.1668 | 1.3583 | 1287.9181 | 0.074473 | 6.4899 | 0.73622 |
| 88 | -0.32674 | 0.040472 | 1.21 | 156.5058 | -0.039474 | 5.0906 | 0.82646 |
| 89 | 0.66763 | -0.18838 | 1.1524 | 225.6066 | 0.42191 | 4.1392 | 0.86775 |
| 90 | -0.048017 | 0.75259 | 1.2052 | 114.4079 | -0.049822 | 5.0127 | 0.82971 |
| 91 | -0.95871 | 1.0441 | 1.4426 | 187.0423 | -0.95159 | 11.0008 | 0.69318 |
| 92 | -0.095613 | -0.04626 | 1.3895 | 566.3438 | -0.080484 | 4.9155 | 0.71968 |
| 93 | -0.093959 | 0.045828 | 1.2414 | 250.1733 | 0.0155 | 7.6338 | 0.80556 |
| 94 | -0.21889 | -0.058404 | 1.2653 | 716.119 | -0.17457 | 4.3766 | 0.7903 |
| 95 | 0.17654 | 0.1917 | 1.2213 | 69.8449 | 0.17572 | 5.2528 | 0.81883 |
| 96 | -0.25315 | 0.45417 | 1.499 | 65.9894 | -0.058384 | 15.3853 | 0.66711 |
| 97 | 0.00061051 | 0.51783 | 1.2351 | 25.0439 | 0.012296 | 7.3517 | 0.80968 |
| 98 | 0.003033 | -0.12126 | 1.2362 | 161.6077 | 0.20659 | 5.9214 | 0.80894 |
| 99 | 0.13514 | 0.083569 | 1.2585 | 514.4767 | 0.47655 | 6.1469 | 0.79461 |
| 100 | -0.15465 | -0.040802 | 1.3458 | 226.0653 | -0.10702 | 5.4115 | 0.74304 |
| 101 | -0.11147 | -0.32719 | 1.33 | 54.4961 | -0.090004 | 6.0987 | 0.75188 |
| 102 | -0.13052 | -0.10792 | 1.3203 | 968.932 | -0.035882 | 5.4075 | 0.75739 |
| 103 | 0.067141 | -0.099938 | 1.2758 | 43.9797 | 0.066755 | 4.4161 | 0.78385 |
| 104 | 0.041719 | -0.17166 | 1.6498 | 251.914 | 0.17371 | 7.5275 | 0.60612 |
| 105 | -0.16835 | -0.015715 | 1.7548 | 1317.8853 | -0.12918 | 7.7906 | 0.56987 |
| 106 | -0.28082 | 0.34674 | 1.4312 | 752.5936 | 0.143 | 4.7783 | 0.69871 |
| 107 | -0.077993 | 0.79769 | 1.2693 | 95.8903 | 0.065384 | 9.4025 | 0.78784 |
| 108 | -0.34712 | 0.11838 | 1.2639 | 923.4769 | -0.21134 | 5.5944 | 0.7912 |
| 109 | 0.061508 | 0.69907 | 1.185 | 211.5988 | 0.45093 | 7.5936 | 0.8439 |
| 110 | 0.1764 | -0.019276 | 1.2477 | 371.369 | -0.028347 | 6.189 | 0.80146 |
| 111 | -0.78324 | 0.47637 | 1.5137 | 499.4588 | -0.58253 | 8.9615 | 0.66064 |
| 112 | -0.28061 | 0.25703 | 1.2318 | 341.1666 | -0.43298 | 6.8023 | 0.81179 |

| Patient | Shape_compactness2_AIP | LoG_sigma_3_mm_3D_glcm_infoCorr2_AIP | Wavelet_HLL_stats_skewness_AIP | Wavelet_LLL_stats_max_AIP | LoG_sigma_3_mm_3D_glcm_correl1_AIP | Wavelet_LHH_glcm_correl1_AIP | Wavelet_HLH_rlgl_lowGrayLevelRunEmphasis_AIP |
|---|---|---|---|---|---|---|---|
| 1 | 0.32549 | 0.64039 | -0.39901 | -716.8853 | 0.52468 | 0.31126 | 0.0091597 |
| 2 | 0.5347 | 0.61052 | -0.098426 | 51.4881 | 0.58744 | 0.24129 | 0.0041007 |
| 3 | 0.40267 | 0.69073 | 0.053624 | -434.2338 | 0.65327 | 0.27896 | 0.0055794 |
| 4 | 0.39388 | 0.75049 | -0.57803 | 951.8377 | 0.65507 | 0.27448 | 0.0012172 |
| 5 | 0.1933 | 0.73456 | -0.25616 | 188.0641 | 0.62682 | 0.2501 | 0.0030743 |
| 6 | 0.45799 | 0.63263 | -0.17162 | 151.8542 | 0.6008 | 0.30662 | 0.0034199 |
| 7 | 0.25653 | 0.68589 | 0.071696 | 159.7135 | 0.65187 | 0.29133 | 0.0050112 |
| 8 | 0.19176 | 0.66757 | -0.63486 | 1176.8007 | 0.53713 | 0.26614 | 0.0036804 |
| 9 | 0.35633 | 0.70872 | -0.94339 | 214.6891 | 0.63614 | 0.24422 | 0.0031774 |
| 10 | 0.2704 | 0.64844 | -0.66713 | 1003.4632 | 0.60613 | 0.17944 | 0.0028821 |
| 11 | 0.38734 | 0.68864 | -1.2701 | 62.759 | 0.65526 | 0.21151 | 0.0045055 |
| 12 | 0.34016 | 0.62603 | -0.17738 | 124.7681 | 0.60467 | 0.21415 | 0.005743 |
| 13 | 0.26775 | 0.68092 | -0.53652 | 230.4795 | 0.56858 | 0.20613 | 0.0053265 |
| 14 | 0.30782 | 0.72121 | -0.12298 | 159.9268 | 0.66649 | 0.35306 | 0.0024697 |
| 15 | 0.462 | 0.68351 | -0.048069 | 130.6948 | 0.61885 | 0.23784 | 0.0058291 |
| 16 | 0.38216 | 0.67097 | -0.1284 | 129.8138 | 0.64911 | 0.32824 | 0.0035984 |
| 17 | 0.32581 | 0.65515 | -0.17632 | 130.5176 | 0.61655 | 0.2273 | 0.0027998 |
| 18 | 0.25337 | 0.7321 | -0.35554 | 411.547 | 0.63356 | 0.29998 | 0.0028188 |
| 19 | 0.39048 | 0.57246 | 0.24398 | 147.8912 | 0.54975 | 0.21608 | 0.0081868 |
| 20 | 0.56415 | 0.66716 | -0.60285 | 140.0369 | 0.6497 | 0.27196 | 0.0056143 |
| 21 | 0.30197 | 0.64215 | -0.5247 | 126.9844 | 0.61919 | 0.20553 | 0.0059907 |
| 22 | 0.31424 | 0.58523 | -0.36777 | 140.1707 | 0.53818 | 0.25714 | 0.0037487 |
| 23 | 0.32006 | 0.70728 | -0.24612 | 649.459 | 0.62488 | 0.22298 | 0.0031789 |
| 24 | 0.28168 | 0.65236 | 0.38555 | 152.5415 | 0.62732 | 0.24998 | 0.0033316 |
| 25 | 0.33314 | 0.68844 | -0.45853 | 150.465 | 0.63516 | 0.2834 | 0.0031501 |
| 26 | 0.3916 | 0.64546 | 0.10793 | 127.4363 | 0.59989 | 0.25319 | 0.0039353 |
| 27 | 0.28214 | 0.64451 | -0.4085 | 164.6941 | 0.61969 | 0.24392 | 0.0046847 |
| 28 | 0.34727 | 0.67545 | 0.34229 | 143.1662 | 0.60171 | 0.32826 | 0.0035177 |
| 29 | 0.36454 | 0.70728 | -0.78105 | 213.9985 | 0.66416 | 0.26989 | 0.0028684 |
| 30 | 0.21718 | 0.62585 | 0.025625 | 202.2539 | 0.59431 | 0.26135 | 0.0051934 |
| 31 | 0.48737 | 0.64232 | -0.093749 | 159.9245 | 0.55923 | 0.29433 | 0.0048353 |
| 32 | 0.50049 | 0.61485 | -0.61775 | -406.9511 | 0.59935 | 0.22378 | 0.0066998 |
| 33 | 0.48184 | 0.68773 | 0.25456 | 148.8944 | 0.65728 | 0.29995 | 0.0029007 |
| 34 | 0.52267 | 0.69169 | -0.26088 | -76.4537 | 0.66425 | 0.29604 | 0.0042551 |
| 35 | 0.4187 | 0.64417 | -0.1347 | 376.339 | 0.55608 | 0.23053 | 0.0044251 |
| 36 | 0.3996 | 0.61845 | -0.062079 | 151.8335 | 0.5544 | 0.21199 | 0.0060896 |
| 37 | 0.36313 | 0.71413 | -0.43186 | 146.4486 | 0.60674 | 0.32092 | 0.0041293 |
| 38 | 0.29571 | 0.67886 | -1.4021 | 540.2164 | 0.64328 | 0.24618 | 0.0035434 |
| 39 | 0.31057 | 0.70363 | -0.3721 | 1046.9842 | 0.65806 | 0.27647 | 0.001442 |

| | | | | | | | |
|---|---|---|---|---|---|---|---|
| 40 | 0.39749 | 0.674 | -0.077575 | -97.5487 | 0.66314 | 0.23837 | 0.0057826 |
| 41 | 0.4342 | 0.65182 | -0.10093 | 113.7405 | 0.63689 | 0.21107 | 0.0043723 |
| 42 | 0.17812 | 0.5826 | 0.044683 | 110.7841 | 0.56689 | 0.22396 | 0.0040212 |
| 43 | 0.46494 | 0.63135 | 0.090927 | 91.6757 | 0.59859 | 0.2509 | 0.009061 |
| 44 | 0.28678 | 0.64106 | -0.62554 | 154.5605 | 0.58197 | 0.27945 | 0.0039411 |
| 45 | 0.33105 | 0.75346 | -0.20433 | 213.8476 | 0.62751 | 0.24973 | 0.0029509 |
| 46 | 0.44308 | 0.60705 | 0.17296 | 86.5835 | 0.55002 | 0.21827 | 0.0059219 |
| 47 | 0.38764 | 0.67313 | -0.2882 | 159.3977 | 0.61882 | 0.27976 | 0.0036371 |
| 48 | 0.33773 | 0.61308 | 0.17044 | 78.6317 | 0.59435 | 0.24704 | 0.0055401 |
| 49 | 0.2601 | 0.69448 | -0.38185 | 223.9231 | 0.64877 | 0.27151 | 0.0025684 |
| 50 | 0.36783 | 0.59016 | 0.047239 | 163.9244 | 0.57285 | 0.2115 | 0.005449 |
| 51 | 0.3181 | 0.68505 | -0.69265 | 274.0241 | 0.629 | 0.2732 | 0.0025462 |
| 52 | 0.43292 | 0.67293 | -0.18186 | -18.5046 | 0.56894 | 0.27893 | 0.0043674 |
| 53 | 0.5825 | 0.67247 | 0.61147 | 149.694 | 0.52817 | 0.18518 | 0.0056731 |
| 54 | 0.30937 | 0.6276 | -0.36972 | 204.3488 | 0.61652 | 0.16579 | 0.0039766 |
| 55 | 0.36082 | 0.67749 | 0.24671 | 60.0253 | 0.63816 | 0.23878 | 0.004331 |
| 56 | 0.28555 | 0.68979 | 0.10596 | 241.8853 | 0.66045 | 0.28022 | 0.0030412 |
| 57 | 0.4574 | 0.7921 | 0.36271 | 218.5182 | 0.6843 | 0.30785 | 0.0055785 |
| 58 | 0.37507 | 0.58132 | 0.23066 | 131.9748 | 0.57108 | 0.21124 | 0.0046072 |
| 59 | 0.41463 | 0.66076 | -0.0062164 | 192.5715 | 0.61333 | 0.24936 | 0.0032773 |
| 60 | 0.56069 | 0.68681 | 0.41728 | 132.8399 | 0.64689 | 0.27823 | 0.0031086 |
| 61 | 0.55171 | 0.7037 | 0.12732 | 53.375 | 0.55567 | 0.32236 | 0.015033 |
| 62 | 0.30111 | 0.60583 | -0.19592 | -17.5214 | 0.59431 | 0.21493 | 0.0030806 |
| 63 | 0.47945 | 0.65637 | 0.28476 | 132.5173 | 0.49601 | 0.29741 | 0.0061803 |
| 64 | 0.41548 | 0.7284 | 0.021 | 196.7053 | 0.67973 | 0.36635 | 0.0065707 |
| 65 | 0.46449 | 0.64718 | -0.067326 | 33.2827 | 0.61207 | 0.24714 | 0.0055626 |
| 66 | 0.34951 | 0.60907 | -1.2985 | 157.2674 | 0.60512 | 0.17818 | 0.011922 |
| 67 | 0.32058 | 0.72729 | -0.46452 | 181.3909 | 0.68192 | 0.27385 | 0.003273 |
| 68 | 0.21231 | 0.61291 | -0.033839 | 158.6916 | 0.5703 | 0.21129 | 0.0040108 |
| 69 | 0.43964 | 0.62683 | -0.081335 | 70.4818 | 0.58128 | 0.28383 | 0.0056899 |
| 70 | 0.38954 | 0.66544 | 0.072377 | 250.8889 | 0.65816 | 0.21512 | 0.0028924 |
| 71 | 0.20578 | 0.65551 | -0.28696 | 249.9265 | 0.54699 | 0.30885 | 0.0062683 |
| 72 | 0.46625 | 0.62028 | -0.40084 | -219.6804 | 0.60677 | 0.25876 | 0.0046986 |
| 73 | 0.42847 | 0.67855 | 0.10026 | 219.905 | 0.62961 | 0.27038 | 0.003761 |
| 74 | 0.37367 | 0.70068 | -0.22092 | 464.2734 | 0.69075 | 0.22598 | 0.0018045 |
| 75 | 0.3801 | 0.62672 | -0.017451 | 149.6935 | 0.59699 | 0.20499 | 0.0028889 |
| 76 | 0.48621 | 0.64879 | -0.24513 | 224.2238 | 0.60876 | 0.23066 | 0.005718 |
| 77 | 0.53665 | 0.62852 | -0.20999 | 156.1936 | 0.4613 | 0.29548 | 0.0068394 |
| 78 | 0.57054 | 0.68937 | 0.099734 | 131.3334 | 0.60614 | 0.22433 | 0.0087718 |
| 79 | 0.53281 | 0.6468 | 0.38138 | 152.0611 | 0.58792 | 0.30962 | 0.0042015 |
| 80 | 0.4716 | 0.72159 | -0.24775 | 245.856 | 0.68676 | 0.24886 | 0.0045719 |

| | | | | | | | |
|---|---|---|---|---|---|---|---|
| 81 | 0.61722 | 0.57294 | -0.39661 | 158.3339 | 0.54259 | 0.2345 | 0.012543 |
| 82 | 0.55213 | 0.71295 | 0.36588 | 285.3506 | 0.47513 | 0.24819 | 0.0042013 |
| 83 | 0.51176 | 0.67961 | -0.063232 | 50.7258 | 0.61763 | 0.26789 | 0.006145 |
| 84 | 0.50531 | 0.57077 | 0.36214 | 152.2802 | 0.53084 | 0.20476 | 0.010263 |
| 85 | 0.47408 | 0.7458 | -0.29246 | 189.416 | 0.67036 | 0.33355 | 0.0044674 |
| 86 | 0.24341 | 0.71691 | 0.18237 | 392.5526 | 0.64008 | 0.26428 | 0.0012727 |
| 87 | 0.39905 | 0.66553 | 0.21843 | 135.985 | 0.56892 | 0.27567 | 0.0041248 |
| 88 | 0.56451 | 0.62524 | 0.46069 | 10.1431 | 0.57766 | 0.28973 | 0.0062372 |
| 89 | 0.65342 | 0.77344 | -0.98668 | -144.11 | 0.59895 | 0.27942 | 0.016075 |
| 90 | 0.57119 | 0.68149 | -0.19019 | 78.7716 | 0.6289 | 0.31489 | 0.014737 |
| 91 | 0.33307 | 0.59071 | 0.10952 | 723.8474 | 0.55756 | 0.23525 | 0.0035142 |
| 92 | 0.37275 | 0.64374 | 0.42264 | 136.0577 | 0.60084 | 0.31758 | 0.0035542 |
| 93 | 0.52274 | 0.61791 | -0.15615 | 170.5241 | 0.57264 | 0.26224 | 0.0041046 |
| 94 | 0.4936 | 0.62736 | -0.43789 | -130.9008 | 0.54154 | 0.29596 | 0.010343 |
| 95 | 0.54901 | 0.64151 | -0.069381 | 111.3562 | 0.62091 | 0.21621 | 0.0045712 |
| 96 | 0.29689 | 0.59101 | -0.29956 | 427.4618 | 0.54924 | 0.2401 | 0.0024809 |
| 97 | 0.53081 | 0.63875 | 0.47833 | 133.5248 | 0.62784 | 0.18613 | 0.0073544 |
| 98 | 0.52935 | 0.63024 | 0.50317 | 108.4008 | 0.58412 | 0.29418 | 0.0058849 |
| 99 | 0.50172 | 0.70086 | -0.054408 | 270.6781 | 0.58514 | 0.27907 | 0.0048516 |
| 100 | 0.41024 | 0.63089 | -0.33729 | 189.5471 | 0.50845 | 0.29269 | 0.0052238 |
| 101 | 0.42506 | 0.64841 | 0.25767 | 96.0867 | 0.63082 | 0.25415 | 0.0050032 |
| 102 | 0.43448 | 0.64705 | -0.19908 | 191.9445 | 0.55662 | 0.2532 | 0.0037413 |
| 103 | 0.48161 | 0.6514 | 0.07329 | 181.874 | 0.62904 | 0.20142 | 0.0050399 |
| 104 | 0.22268 | 0.68866 | -0.17084 | 196.2886 | 0.63115 | 0.2971 | 0.0041524 |
| 105 | 0.18506 | 0.64556 | -0.19042 | 264.7257 | 0.62356 | 0.26031 | 0.0024121 |
| 106 | 0.34111 | 0.66286 | -0.076798 | 203.0279 | 0.58327 | 0.29752 | 0.0048768 |
| 107 | 0.489 | 0.63087 | 0.62271 | 163.14 | 0.60162 | 0.2634 | 0.0048432 |
| 108 | 0.4953 | 0.67346 | -0.35145 | 166.9744 | 0.52395 | 0.19341 | 0.0062597 |
| 109 | 0.60099 | 0.67877 | 0.67138 | 197.6163 | 0.58857 | 0.32086 | 0.0045459 |
| 110 | 0.5148 | 0.64049 | -0.51329 | -561.4378 | 0.56373 | 0.24984 | 0.01088 |
| 111 | 0.28833 | 0.63317 | 0.49212 | 262.4335 | 0.54177 | 0.2454 | 0.0036398 |
| 112 | 0.53497 | 0.6207 | 0.13217 | 198.8119 | 0.51248 | 0.25475 | 0.0048612 |
