# Supplementary material for: Associations of Radiomic Data Extracted from Static and Respiratory-Gated CT Scans with Disease Recurrence in Lung Cancer Patients Treated with SBRT
Source: PLoS One. 2017 Jan 3;12(1):e0169172. doi: 10.1371/journal.pone.0169172 (PMC5207741; doi:10.1371/journal.pone.0169172)
Supplement: S1 Table — (PDF) [file pone.0169172.s008.pdf]

S1 Table. Description of selected radiomic features

Feature	Group	Description
Sphericity	Shape	How close the tumor is to a sphere with similar volume
Sphere disproportionality	Shape	How far the tumor is to a sphere with similar volume
Compactness	Shape	How compact the tumor is (low area/volume ratio)
Max	Statistics	Max voxel intensity value
Min	Statistics	Min voxel intensity value
Mean	Statistics	Mean voxel intensity value
Range	Statistics	The range of voxels intensity values
Skewness	Statistics	Describe the shape of a probability distribution of the voxel intensity histogram
Total energy	Statistics	Describe the energy of the image
Variance	Statistics	Variance of the voxel intensity histogram
Kurtosis	Statistics	Describe the shape of a probability distribution of the voxel intensity histogram
GLCM homogeneity	Texture	Sensitive to similar patterns of voxels across the whole tumor
GLCM correl	Texture	Correlation of the GLCM matrix
GLCM infoCorr	Texture	Generalization of the classical correlation coefficient of a normal distribution
GLCM clusProm	Texture	Sensitive to flat zones (areas of connecting voxels with the same value)
GLSZM high intensity large area emphasis	Texture	Sensitive to flat zones with high intensity voxels
GLCM clusShade	Texture	Sensitive to flat zones
RLGL low gray level run emphasis	Texture	Sensitive to low intensity heterogeneity
GLSZM large area emphasis	Texture	Sensitive to large flat zones

*Some of these features have Wavelet and Laplacian of Gaussian filters applied which enhance the fine and coarse textures of the tumor
