# Supplementary material for: Associations of Radiomic Data Extracted from Static and Respiratory-Gated CT Scans with Disease Recurrence in Lung Cancer Patients Treated with SBRT
Source: PLoS One. 2017 Jan 3;12(1):e0169172. doi: 10.1371/journal.pone.0169172 (PMC5207741; doi:10.1371/journal.pone.0169172)
Supplement: S2 Table — (PDF) [file pone.0169172.s009.pdf]

S2 Table. Spearman's correlation coefficient of strongly correlated FB and AIP imaging features

FB feature	FB feature group	AIP feature	AIP feature group	ρ
LoG 3mm 3D GLCM homogeneity1	Texture	LoG 3mm 3D GLCM homogeneity1	Texture	0.87
Wv HLH GLSZM highIntensityLargeAreaEmp	Texture	Wv LHH GLSZM large area emphasis	Texture	0.92
Volume	Shape	Wv LHH GLSZM large area emphasis	Texture	0.82
Sphere disproportionality	Shape	Sphere disproportionality	Shape	0.86
Sphericity	Shape	Sphericity	Shape	0.85
Sphericity	Shape	Compactness2	Shape	0.84
Max. diameter	Shape	Max. diameter	Shape	0.97
Max. diameter	Shape	Volume	Shape	0.81
Volume	Shape	Volume	Shape	0.97
Sphericity	Shape	Sphere disproportionality	Shape	-0.86
Sphere disproportionality	Shape	Sphericity	Shape	-0.84
Sphere disproportionality	Shape	Compactness2	Shape	-0.82

Labels: Wv = wavelet; LoG = Laplacian of Gaussian; L = low; H = high; GLCM = Gray-Level Co-occurrence Matrix; GLSZM = Gray-Level Size Zone Matrix; RLGL = Run Low Gray Level; ρ = Spearman's correlation coefficient
