# Supplementary material for: Associations of Radiomic Data Extracted from Static and Respiratory-Gated CT Scans with Disease Recurrence in Lung Cancer Patients Treated with SBRT
Source: PLoS One. 2017 Jan 3;12(1):e0169172. doi: 10.1371/journal.pone.0169172 (PMC5207741; doi:10.1371/journal.pone.0169172)
Supplement: S3 Table — (PDF) [file pone.0169172.s010.pdf]

S3 Table. Concordance indices of significant AIP imaging features

	Imaging feature	Feature Group	C-index	p-value*
Distant metastasis (DM)	Max. diameter	Conventional	0.658	0.015
	Volume	Conventional	0.643	0.022
	Wv HLH RLGL low gray level run emphasis	Texture	0.676	0.0088
	GLCM correl1	Texture	0.649	0.014
	Sphericity	Shape	0.648	0.0088
	Sphere disproportionality	Shape	0.648	0.0088
	Sphere compactness2	Shape	0.648	0.0088
	LoG 3mm 3D GLCM correl1	Texture	0.648	0.015
	Wv HLL skewness	Statistics	0.638	0.031

Labels: Wv = wavelet; LoG = Laplacian of Gaussian; L = low; H = high; GLCM = Gray-Level Co-occurrence Matrix; RLGL = Run Low Gray Level; stats = statistics; C-index = concordance index; *p-values were false discovery rate corrected
